# Comprehensive Comparisons between Grafted Kynam Agarwood and Normal Agarwood on Traits, Composition, and In Vitro Activation of AMPK

**DOI:** 10.3390/molecules28041667

**Published:** 2023-02-09

**Authors:** Fengming Chen, Yu Huang, Lu Luo, Qiaochu Wang, Nanxi Huang, Zhijie Zhang, Zhen Li

**Affiliations:** 1 Institute of Chinese Materia Medica, China Academy of Chinese Medical Sciences, Beijing 100700, China; 2 MOE Key Laboratory of Bioinformatics, School of Life Sciences, Tsinghua University, Beijing 100084, China; 3 Georgetown University Medical Center, Washington, DC 20057, USA

**Keywords:** Grafted Kynam agarwood, *Aquilaria sinensis*, MS-based high-throughput analysis, Molecular docking, Flindersia-type 2-(2-phenylethyl) chromones, AMPK

## Abstract

Agarwood, a highly valuable resin/wood combination with diverse pharmacological activities but scarce supply, has a long history of being used as a medicine in several medical systems. Grafted Kynam agarwood (GKA) has been cultivated successfully recently and has the qualities meeting the definition of premium Kynam agarwood. However, there are few comprehensive comparisons between GKA and normal agarwood in terms of traits, global composition, and activity, and some key issues for GKA to be adopted into the traditional Chinese medical (TCM) system have not been elaborated. The two types of agarwood samples were evaluated in terms of trait characteristics, physicochemical indicators, key component groups, and global compositional profile. Furthermore, a molecular docking was performed to investigate the active ingredients. In vitro activity assays were performed to evaluate the activation of adenosine 5’-monophosphate (AMP)-activated protein kinase (AMPK) by GKA and normal agarwood. The results revealed that, overall, the traits, microscopic characteristics, chemical composition types, and bioactivity between GKA and normal agarwood were similar. The main differences were the content of resin (ethanolic extract content), the content of key component groups, and the composition of the different parent structural groups of 2-(2-phenethyl) chromones (PECs). The contents of total PEC and ethanol extract content of GKA were significantly higher than those of normal agarwood. The MS-based high-throughput analysis revealed that GKA has higher concentrations of sesquiterpenes and flindersia-type 2-(2-phenylethyl) chromones (FTPECs) (m/z 250-312) than normal agarwood. Molecular docking revealed that parent structural groups of FTPECs activated multiple signaling pathways, including the AMPK pathway, suggesting that FTPECs are major active components in GKA. The aim of this paper is to describe the intrinsic reasons for GKA as a high-quality agarwood and a potential source for novel drug development. We combined high-throughput mass spectrometry and multivariate statistical analysis to infer the different components of the two types of agarwood. Then we combined virtual screening and in vitro activity to construct a component/pharmacodynamic relationship to explore the causes of the activity differences between agarwood with different levels of quality and to identify potentially valuable lead compounds. This strategy can also be used for the comprehensive study of other TCMs with different qualities.

## 1. Introduction

Agarwood is a valuable resin/wood combination. Resin is produced by a variety of plants in two genera, *Aquilaria* spp. and *Gyrinops* spp., in response to stimuli from external damage to help the plant heal itself [1,2]. Those stimuli from natural or artificial sources (knife, fire, fungus, etc.) put the Aquilaria tree in a state of stress and cause it to produce a special defensive mechanism to secrete resin [3]. The majority of available agarwood in the market is from four species, including *Aquilaria malaccensis* Lam, *Aquilaria crassna* Pierre ex Lecomte, *Aquilaria sinensis* (Lour.) Gilg, and *Aquilaria filaria* (Oken) Merr [4]. Natural agarwood has been used as incense and medicine for spiritual healing and physical healing. The therapeutic effects of agarwood are closely related to its quality with high quality agarwood containing higher concentrations of resins [3,5]. Agarwood has been included in every edition of the Pharmacopoeia of the People’s Republic of China (Volume 1) (Ch.P) [6]. Although both domestic agarwood (*Aquilaria sinensis* (Lour.) Gilg) and imported agarwood (*Aquilaria agallocha* Roxb.) were included in the previous edition of Ch.P (1963 edition), only *Aquilaria sinensis* (Lour.) Gilg has been included in subsequent editions of Ch.P as the designated medicinal agarwood resource. It is hard for plants forming agarwood under natural condition, which results in the low supply of agarwood. With the demand outstripping the supply, natural agarwood resources are in danger of being depleted. Both *Aquilaria* spp. and *Gyrinops* spp. have been listed in Appendix II of the Convention on International Trade in Endangered Species of Wild Fauna and Flora (CITES).

Kynam agarwood, a rare high-quality agarwood, also known as Kanankoh, Kyara, Kynam, Chi-Nan, Qi-Nan, and so on, has consistent characteristics of high resin content, soft texture, and unique aroma [7,8]. Because of the depletion of natural Kynam agarwood, Kynam agarwood is expensive and out of reach of the general public. Fortunately, recently developed artificial cultivation techniques can produce Kynam agarwood with native *Aquilaria sinensis,* referred as grafted Kynam agarwood (GKA). Some studies including germplasm genetics, incense smoke, and chemical composition studies have shown that GKA has the potential to become a high-quality agarwood. Yong Kang et al. performed DNA barcoding analysis on 58 batches of GKA in the market and found that it was closely related to A. sinensis in the phylogenetic tree and therefore tentatively identified its source species as A. sinensis [9]. Yuan Chen et al. concluded that phenylethyl chromones with no or few substituents and sesquiterpenes in GKA were the main factors contributing to its aroma by comparing incense smoke analysis from GKA and normal agarwood [10]. Meng Yu et al. suggested that the main differences between the GKA and normal agarwood were 2-(2-phenylethyl) chromone and 2-[2-(4’-methoxybenzene) ethyl] chromone, both of which were higher in GKA than in common agarwood [11]. Feng Jian et al. conducted a full inspection of ten batches of GKA collected according to the 2020 edition of Ch.P and found that agarotetrol content did not meet the requirement of 0.1% in pharmacopoeia, but the ethanol extract content of GKA was much higher than the requirement of 10% in the pharmacopoeia, and the characteristics were consistent with the profiles of high-quality agarwood [12]. Wen-Yi Kao et al. analyzed the chemical composition of the incense smoke produced from four agarwood including Kynam by headspace gas chromatography/tandem mass spectrometry and found that 2-(2-phenylethyl) chromone derivatives were only found in the incense smoke produced from Kynam agarwood [13]. Chen xiqin et al. investigated the in vitro anti-inflammatory activity of the essential oil extracts of Kynam and normal agarwood based on the LPS-induced RAW264.7 cell model and found that the essential oil extracts of Kynam agarwood had better anti-inflammatory activity [14]. Despite the great efforts of these studies on the multi-component analysis of GKA and normal agarwood, more evaluations of GKA are required to further understand its pharmacological and therapeutic effects. Moreover, there are few studies studying the effects and mechanism of GKA at the molecular level and the downstream signaling pathways that are influenced by GKA, which gives doctors and regulators limited references for regulations. Study of GKA characteristic components, biosynthetic pathways, and activities is important for the understanding and expansion of new sources of medicinal agarwood.

Flindersia-type 2-(2-phenylethyl) chromones (FTPECs) are considered to be an important group of active ingredients in agarwood [15,16,17,18]. Previous studies have identified that FTPECs have a variety of activities such as anti-inflammatory [19,20,21,22], AChE inhibition [23,24,25], antioxidant [26], cytotoxic [27,28,29], PPARγ partial agonist [30], tyrosinase inhibition [31], neuroprotection [32,33], α-glucosidase inhibition [34], and phosphodiesterase (PDE) 3A and 5A1 inhibitory activities [35,36]. Several studies have revealed that FTPECs exhibit different biological properties through different substituents and positions [18,37]. For example, FTPECs containing 3’-hydroxy and 4’-methoxy substituents showed stronger AChE inhibitory activity than other compounds [23]. Therefore, there is an increasing interest in studying FTPECs.

One pathway that can be influenced by agarwood is AMPK. AMPK is a serine/threonine kinase, a heterotrimer composed of a catalytic α subunit and regulatory β and γ subunits, that acts as a central sensor of cellular energy status. AMPK has been found to be an important drug target for a variety of diseases such as diabetes. Metformin is an activator of AMPK and is used as the first-line clinical treatment for type 2 diabetes [38]. Recent studies have also found that regulation of the AMPK signaling pathway may be effective in inhibiting the development and progression of myocardial hypertrophy and heart failure [39]. Activation of AMPK has been considered for the treatment of chronic inflammatory diseases [40]. AMPK activators have also been found to promote apoptosis by inhibiting cell proliferation and activating AMPK phosphorylation, implying that AMPK agonists can function as anticancer agents [41,42].

It is difficult to acquire monomer chemicals from secondary metabolites due to the similarity of parent nucleus structures and the difficulty of separation, and the alternative of obtaining them using chemical synthesis is an equally time-consuming and costly process. Screening approaches can speed the research pace through computational tools combined with the integrity of network analysis, which is especially useful to evaluate the “multi-component, multi-channel, multi-target” synergy of herbal medicines [43,44]. For example, recent studies identified metalloestrogens and phytochemical estrogens that can interact with estrogen receptors and disrupt estrogen regulated signaling pathways [45,46,47]. With the molecular docking, potential metals and phytochemicals were identified and helped narrow the number of candidates for in vivo or in vitro assays. This method can also be applied to traditional medicine studies, which can help reduce the workload and identify the potential candidates in an effective and efficient manner. Others used the similar method to explore the effective components and mechanism of Polygonati Rhizoma in the treatment of osteoporosis, which provided a pharmacological mechanism of Polygonati Rhizoma [48].

In this study, we investigated the difference between GKA and normal agarwood on traits, composition, and in vitro activation of AMPK based on an integration strategy. The aim of this paper is to illustrate the intrinsic reasons for GKA as a high-quality agarwood and a potential source for novel drug development through the advantages of the composition and activation of key targets of activity. We combined multiple approaches to analyze the similarities and differences between GAK and normal agarwood from multiple aspects of phytomedicine to provide a comprehensive reference for clinical as well as R&D developers in the use of GKA.

## 2. Results and Discussion

### 2.1. Sample Characteristics and Microscopic Inspection

**Figure 1:**
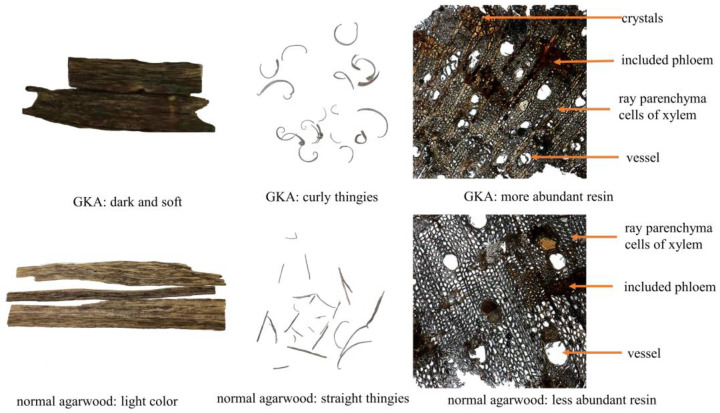
Morphology of the samples of two representative types of agarwood as well as their transverse section microscopic features (magnification of 100×) and wood flake morphology.

In traditional Chinese medicine, medicinal agarwood is graded according to its form, fragrance, and texture. The flakes of GKA has a self-rolling feature and GKA has a very elegant fragrance, as described in traditional Chinese medical documents [8]. Figure 1 showed the morphology of representative slice samples of GKA and normal agarwood under microscope. The microscopic features of the two types of agarwood samples were similar, as shown in Figure 1. The resin secreted in both agarwood types were mainly distributed in ray parenchyma cells of xylem, including phloem and plant catheters, while the main difference was the more abundant resin, indicated by the darker color, and the calcium oxalate crystals in the included phloem of GKA. These crystals may have assisting roles in the agarwood-forming process of the tree, which requires further investigation.

### 2.2. Analysis of Physicochemical Indicators and Content of Key Component Groups

**Figure 2:**
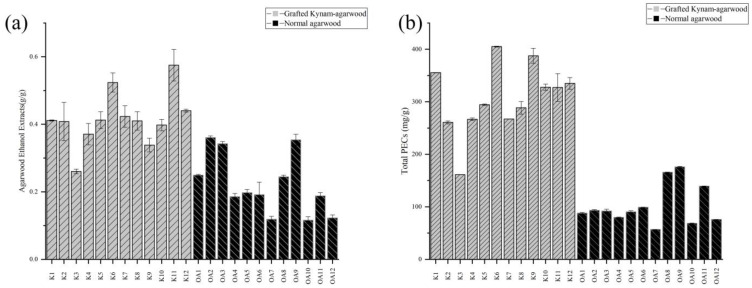
Content of agarwood ethanol extracts (**a**) and total PECs (**b**). The ethanol-soluble extract content was determined according to the procedures of the Chinese Pharmacopoeia. The total PEC content was measured at 254 nm with agarotetrol as the standard.

Analysis of the ethanol extract content is an important method to evaluate the quality of agarwood. 2-(2-phenylethyl) chromones (PECs), which are rare in plants, are the main contents of the chemical content of the extract. Figure 2 and Table 1 summarizes the experimental data of the agarwood ethanol extract and the total content of PECs. Not only was the total content of in ethanol soluble compounds of GKA significantly higher than that of normal agarwood (*p* value < 0.01), the PEC content of the ethanol extract of GKA was also significantly higher than that of normal agarwood (*p* value < 0.01).

### 2.3. Mass Spectrometric Analysis of Chemical Components in Agarwood and Analysis of Common Components

**Figure 3:**
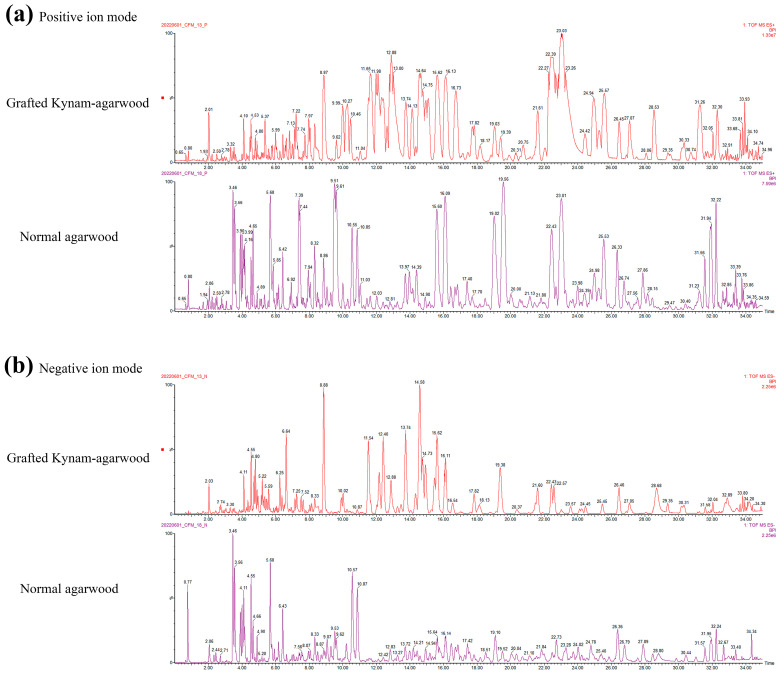
BPI plots of positive ion mode (**a**) and negative ion mode (**b**) of the ethanol extracts of two representative samples of agarwood.

In order to characterize chemical components in the ethanol extracts of the two types of agarwood, their ethanol extracts were subjected to liquid mass analysis under optimized conditions. The positive and negative ion mass spectra of the two types of agarwood extracts are shown in Figure 3. The types of compounds of the two types of agarwood were similar, and their ethanol extracts were mainly composed of 2-(2-phenylethyl) chromones. This is the basis on which both types of agarwood can be used as medicinal agarwood.

The total response values of the peaks were higher for GKA compared with normal agarwood, and the mass peaks of GKA were mainly concentrated in the low polarity part, while those of normal agarwood were mainly concentrated in the high polarity part (e.g., agarotetrol). Overall, 76 components were identified, including 43 FTPECs, 16 THPECs, 3 oxidoagarochromones, and 12 di/trimers, as shown in Table S4. Among the most abundant components of GKA were 2-(2-phenylethyl) chromone, 2-[2-(4-methoxyphenyl) ethyl] chromone, and 2-[2-(3-Hydroxy-4-methoxyphenyl) ethyl] chromone. In summary, the ethanol extracts of the two types of agarwood were similar in composition, with PECs and SESs dominating the compositions. These similar components may contribute to the similar therapeutic effects of two samples. However, GKA has higher concentrations of FTPECs. Normal agarwood has higher concentrations of THPECs. These differences in the parent nucleus structure, substituents, and contents may contribute to the different efficacies. 

### 2.4. Mass Spectrometry-Based High-Throughput Techniques for Processing Raw Data of Two Types of Agarwood and Obtaining Global Difference Components

**Figure 4:**
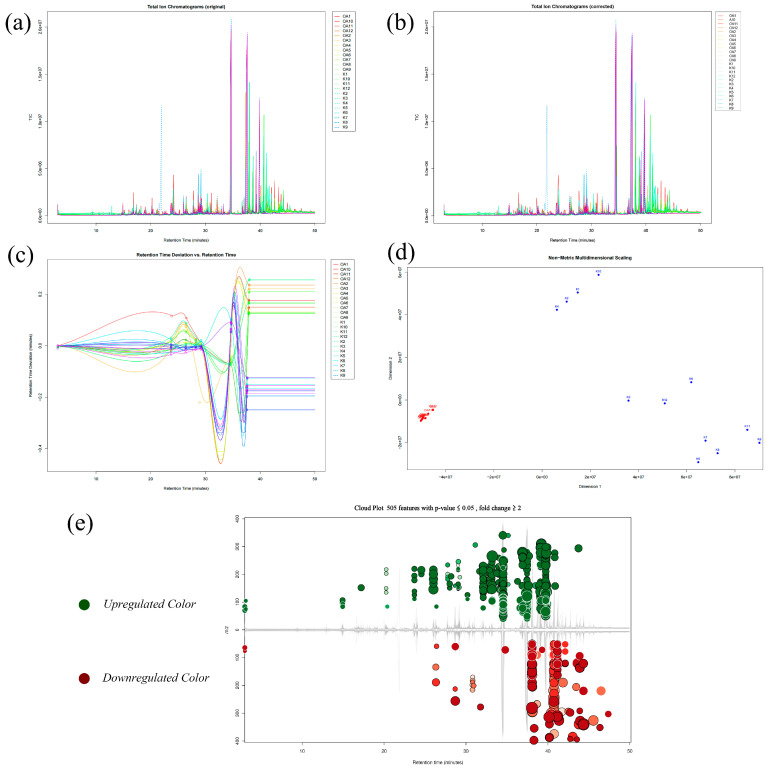
Overview of the results of untargeted GC-MS analysis of the metabolic profiles of all samples by XCMS-Online. The overlap of total ion chromatograms (TIC) before (**a**) and after (**c**) retention time (RT) alignment, the retention time alignment curve (**b**), multidimensional scaling (**d**), and differential feature plot (“mirror plot”) (**e**). The red indicated the features that is more highly expressed in the GKA, and the green represented the opposite. *p* value is represented by how dark or light the color is. Fold change is represented by the radius of each feature.

To further understand the difference between the two groups, we analyzed the metabolic targets. Figure 4 and Figure S1 show an overview of the untargeted metabolic analysis pipeline based on GC-MS and UPLC/TOF-MS, respectively. The key parameter Peakwidth (expected range of chromatographic peakwidth), which is the decisive peak extraction step in untargeted methods based on raw data by mass spectrometry, is evaluated as the suitable extraction range for agarwood sample data by detecting the PECs parent core typical structural compound 2-(2-phenythyl) chromone in the sample, as shown in Figure S2. In data preprocessing, the ANOVA statistical test was used to identify features with high confidence. Based on the data preprocessing methods above, we finally extracted 505,1747,257 candidate features from the raw data obtained by the three analysis methods for further multivariate statistical analysis.

**Figure 5:**
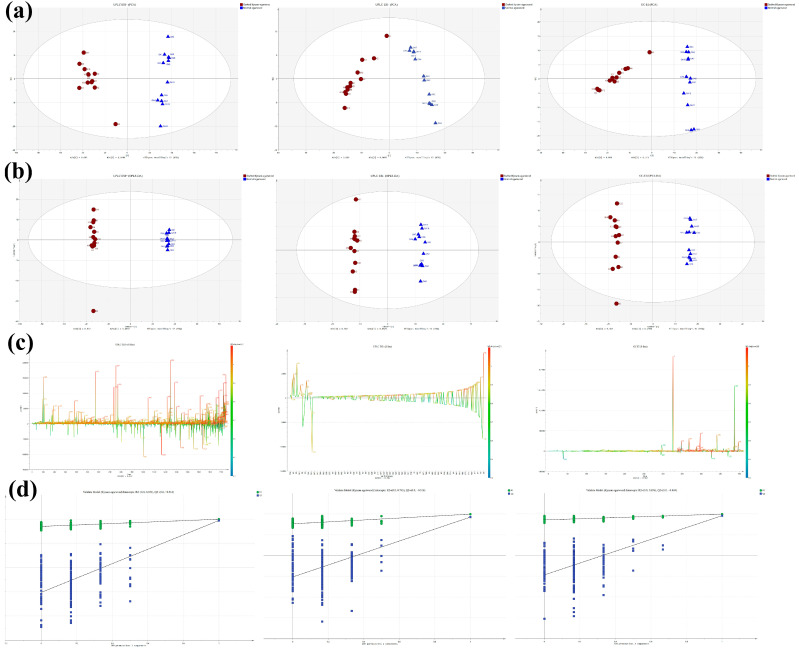
Multivariate statistical analysis revealed metabolic differences between the two types of agarwood. Score plot of the PCA model (**a**), score plot (**b**), S-line plot (**c**), and permutation analysis line plots (**d**) of the OPLS model. The numbers in the S-line plot are the codes for the ion features. The left, middle, and right columns are the multivariate statistical results obtained by GC-MS and UPLC/Q-TOF-MS positive and negative ion modes, respectively.

Multivariate statistical analysis was performed to provide an overall depiction of the differences in metabolomic ionized features between the two agarwood types, as shown in Figure 5. Principal component analysis (PCA) showed that all samples were within the 95% confidence interval, and the GKA and normal agarwood samples were clearly separated, as shown in Figure 5a. Then, OPLS-DA was performed on three sets of data matrices to build classification models and to identify significant differences in metabolic profiles between the two groups of samples (Figure 5b). In the UPLC/TOF-MS positive ion mode and GC-MS results, the K3 sample (The code of the K3 sample corresponds to the code of the samples in Table S1) was outside of the confidence interval, probably because the K3 samples contained more wood-containing parts, and the main effective components of agarwood were in the secreted resin [49].

To evaluate the quality of the model, the R2 and Q2 values were calculated. The three OPLS-DA models in this study had R2X values greater than 0.72, R2Y values greater than 0.99, and Q2 values greater than 0.92. To further distinguish the metabolites associated with different types of agarwood types, an S-line was generated, and the potential biomarkers (VIP value > 1) were displayed with the featureidx (Feature ID) as the label (Figure 5c). Cross-validation and 200 permutation tests were performed to prevent overfitting from occurring during OPLS model development (Figure 5d). Finally, 242, 255, and 70 differential features with high confidence were extracted from the raw data obtained by the three mass spectrometry acquisition methods, respectively.

### 2.5. Identification of Differential Makers between GKA and Normal Agarwood and Differential Analysis of PECs Biosynthesis Pathway 

Based on the high-resolution mass spectral fragment ion information obtained in the positive and negative ion modes of UPLC/Q-TOF-MS, a total of 64 metabolites were identified from positive and negative ion modes, as shown in Table S5. These metabolites included 27 FTPECs, 13 THPECs, 1 epoxy-tetrahydro-2-(2-phenylethyl) chromone (EPEC), 21 sesquiterpenes, and 2 multimers. Currently, natural PECs are only isolated from a few plants, including *Imperata cylindrica* (L.) Beauv and *Aquilaria spp*., and almost all public databases for metabolite identification cannot accurately identify their structures. In this study, we performed a detailed mass fragmentation analysis based on the mass spectra or related reports to confirm the structures of PEC metabolites. The typical representative mass spectra of standard FTPECs (Featureidx: 697, 1125, 981) and standard THPECs (Featureidx: 1039, 1092, 1037) are provided in Figure S3. To identify isomeric compounds without standards, compound structures were inferred from their fragmentation patterns combined with chromatographic retention characteristic. For example, 3 PECs peaks associated with agarwood type discrimination were observed in ESI positive ion mode from their parent ions [M + H] + at m/z 313.1092, 313.1108, 313.1079. Based on the molecular formula and the cleavage properties of PECs, they can be identified as isomers of FTPECs with two hydroxyl groups and one methoxy group. Among them, the high abundance MS fragment ion of two isomers at m/z 121 [M + H − 192] + may indicate that only one methoxy group was connected in the benzyl moiety, and two hydroxyl groups were connected in the chromone moiety, which were deduced as 6,8-dihydroxy-2-[2-(4-methoxy) phenylethyl] chromone and 6,7-dihydroxy-2-[2-(4-methoxy) phenylethyl] chromone based on their chromatographic retention characteristics and related reports. The high abundance MS fragment ion of the other isomer at m/z 137 [M + H − 176] + indicated that the benzyl site contained both a hydroxyl group and a methoxy group, while the other hydroxyl group was located in the chromone site, which can be inferred to be 6-hydroxy-2-[2-(3-hydroxy-4-methoxyl) phenylethyl] chromone. In addition, since sesquiterpenoids in agarwood do not have chemical reference substance and their cleavage rules are complex, the structure of sesquiterpenoids in liquid data cannot be determined.

**Figure 6:**
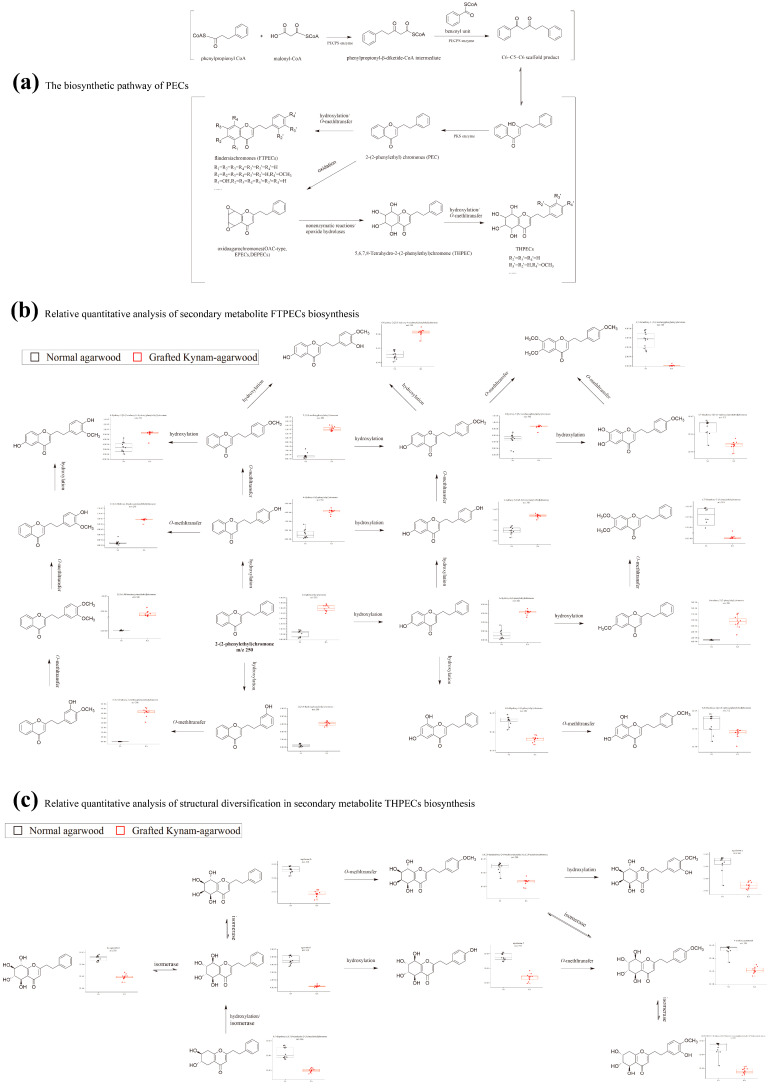
Differential analysis of PECs biosynthesis pathway between GKA and normal agarwood. The biosynthetic pathway of PECs (**a**), relative quantitative analysis of structural diversification in secondary metabolite FTPECs biosynthesis (**b**), relative quantitative analysis of structural diversification in secondary metabolite THPECs biosynthesis (**c**).

The recent discovery by Tu Pengfei’s group revealed part of the biosynthetic pathway of PECs that differs from flavonoids, and a new diarylpentane-producing polyketide synthase (PECPS) from agarwood was considered to be the key enzyme in the synthesis of 2-(2-phenethyl) chromone [50]. PECPS was found to use a one-pot formation mechanism to form C6-C5-C6 scaffolds, the precursor of PEC, as shown in Figure 6a. In contrast to previous understanding [51], recent evidence indicated that FTPECs is the precursor of THPECs and EPECs. Based on the compounds isolated from agarwood, it is speculated that PEC is further modified in biosynthesis, including hydroxylation, methylation, isomerization, epoxidation, halogenation, etc. These post-modification reactions greatly increase the diversity of PECs types and structures. Based on the semi-quantitative data of the ion peak area of MS, we conducted relative quantitative analysis of the expression of 18 FTPECs and 9 THPECs in the biosynthetic pathway, as shown in Figure 6b,c. These results imply that the expression level of key enzymes in the biological pathway of synthesizing FTPECs in GKA was much higher than that of normal agarwood, while normal agarwood has a more complete biosynthetic pathway in the synthesis of relatively complex structures in PECs, such as a few FTPECs with more methoxy substituents in the chromone unit and THPECs. However, higher expression of key enzymes and short pathway may give GKA a greater advantage in enriching FTPECs components.

### 2.6. Identification of Potential Volatile Fragrance Markers in GKA

One important standard to grade medicinal agarwood is fragrance. Agarwood contains two major components, PECs, and sesquiterpenoids, which are considered to be associated with fragrance [52,53]. In terms of the identification of metabolic features acquired by GC-MS, sesquiterpenes and some PECs (such as 2-(2-phenylethyl) chromone) were identified by by the method described in Section 3.6. Table S5 shows the preliminary results of 18 significantly different metabolites, including 12 PECs and 6 SESs, that can be used to distinguish the two types of agarwood from the data collected by GC-MS. The total contents of 2-(2-phenethyl) chromone and 2-[2-(4-methoxyphenyl) ethyl] chromone in GKA were relatively higher (the average proportion was 58.5% in GKA samples), and after heating, the contents are pyrolyzed into benzaldehyde and 4-methoxybenzaldehyde, which is associated with fragrance according to some studies [10]. Other metabolites that was higher in GKA identified in this study are also associated with fragrance. For example, Guaia-1(10),11-dien-15,2-olide has a powerful, long-lasting woody, sweet note [54]. Longifolol has a floral, cedar-wood fragrance and is also popular with industry professionals as a natural resource for modern fragrances [55]. These components may have contributed to the fragrance of GKA and could be used as fragrance marker of GKA.

### 2.7. Molecular Docking Reveals the Major Active Ingredients Group of Agarwood and In Vitro Validation of the Activated AMPK

**Figure 7:**
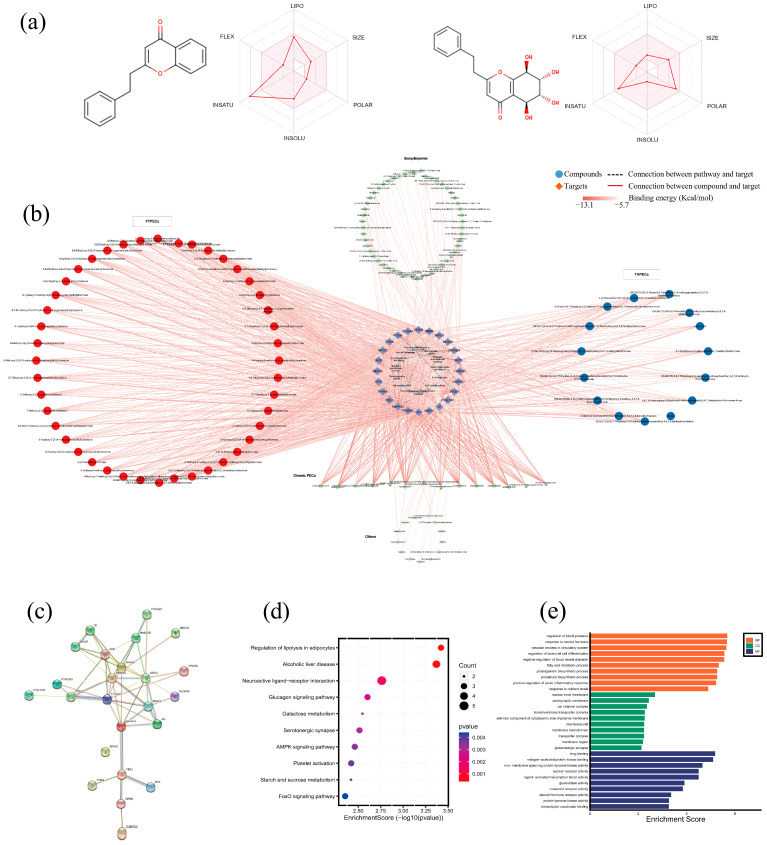
Molecular docking revealed the potential signaling pathways regulated by agarwood related to diseases. The predicted bioavailability radar of 2-(2-phenylethyl) chromone and agarotetrol (**a**), Simulation of a potential target-compound-pathway docking network visualized by Cytoscape (**b**). The depth of the red connecting line between the compound and the target gene represents the result of the free energy of molecular docking. Protein-protein interactions (PPI) of potential targets (**c**), the bubble plot of KEGG pathway enrichment analysis (**d**), and GO analysis of potential targets with top 10 terms displayed in Bar graph (**e**).

Since many failures in synthetic drug development were related to pharmacokinetics, the pharmacokinetics of small molecule compounds in the field of natural products have attracted lots of attention as well as their efficacy and toxicity [56]. Computer models have been used as an effective alternative to experimental procedures for predicting pharmacokinetic parameters, especially selecting targets from large database at initial steps [57]. In this experiment, SwissADME was used to evaluate and screen the ADME parameters of the small molecule compounds library under the optimized condition [58]. Figure 7a showed the predicted bioavailability radar of 2-(2-phenylethyl) chromone, a typical structural compound of FTPECs, and agarotetrol, a typical structural compound of THPECs. The detailed data on the docking scores between different parent nuclear structural compounds and targets displayed in shades of color in the network are shown in Figure 7b and Table S6. The results of ingredient-target interaction prediction revealed that the main active ingredients in the two types of agarwood were PECs. Among them, FTPECs can bind to most screened targets and exhibited a stronger binding ability than THPECs. Taken together, these results suggest that GKA may have stronger therapeutic potential than normal agarwood because of the higher level of FTPEC. The reason that GKA contains a higher quantity and stronger active components may be that it has a shorter biological pathway and higher expression level of key enzymes than that of normal agarwood; therefore, it has a great advantage in accumulating FTPEC components. In addition, there are some dimeric 2-(2-phenylethyl) chromones with very low contents in these two types of agarwood, which exhibit strong affinity for multiple potential targets. Furthermore, deep analysis was performed on the screened genes, including protein-protein interactions (PPI) and KEGG pathway analysis and GO enrichment analysis, as shown in Figure 7c–e. These predicted genes were mainly involved in the regulation of lipolysis in adipocytes, neuroactive ligand-receptor interaction, glucagon signaling pathway, AMPK signaling pathway, etc.

**Figure 8:**
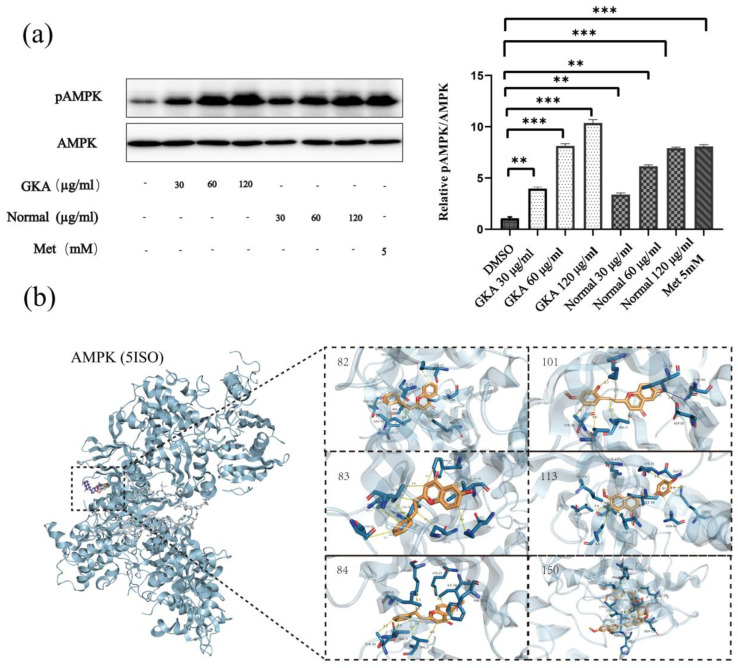
In vitro activity and validation study of the agarwood ethanol extracts activates AMPK in 293T cells. The cell lysates were subjected to SDS-PAGE and Western blot analysis using antibodies specific for phospho-AMPK or total AMPK. The results are presented relative to the vehicle control (DMSO) and as the mean ± SD (*n* = 3). ** *p* < 0.01; *** *p* < 0.001 (**a**). The binding mode of some representative compounds with AMPK; the codes for the compounds in the figure correspond to those in Supplementary Materials Table S2 (**b**).

AMPK was selected to validate the bioactivity of GKA and normal agarwood. Western blot analysis confirmed that at a very low concentration of 30 µg/mL, both agarwood ethanol extracts activated AMPK and significantly increased AMPK phosphorylation in a dose-dependent manner in HEK293T cells (Figure 8a). At the concentration of 120 µg/mL, the treatment of ethanol extract of agarwood resulted in the phosphorylation of AMPK similar to the positive control metformin. The treatment of ethanol extract of GKA results in a greater phosphorylation of AMPK. As we expected, the ethanol extract of GKA exhibited a stronger AMPK activation ability than that of normal agarwood in HEK292T cells. Figure 8b shows a detailed view of the binding mode of some representative compounds in agarwood with high docking scores. Several FTPECs highly expressed in GKA showed high binding energies, such as 2-(2-Phenylethyl) chromone with a docking score of 8.7 and 6-methoxy-2-(2-phenethyl) chromone with a docking score of 8.8 (>7.0 indicates strong binding ability). In a detailed view of the binding pocket, PECs bound protein pockets and interacted with a cluster of hydrophobic residues of each domain, and the polar interactions of polar groups on the chromone moiety and benzyl moiety contributed to activator binding. Furthermore, a previous study reported that FTPECs such as 6-methoxy-2-(2-phenethyl) chromone were partial agonists of peroxisome-proliferator-activated receptor gamma (PPARγ) [30]. PPARγ is a ligand-activated transcription factor that is targeted by antidiabetic drugs of the thiazolidinediones (TZD) class to induce adiponectin-mediated improvement in insulin sensitivity [59]. This implies that FTPECs in GKA may be partial agonists of PPARγ, activators of AMPK, or have a dual ability to activate AMPK/PPARγ. This dual activity may make GKA a good medicinal plant resource for the development of natural products to treat type 2 diabetes and other metabolic syndromes.

## 3. Materials and Methods

### 3.1. Agarwood Materials and Sample Preparation

The 12 normal agarwood (*Aquilaria sinensis* (Lour.) Gilg) and 12 GKA samples were obtained from two main producing areas, namely, Guangdong (20°13′–25°31′ N, 109°39′–117°19′ E) and Hainan (3°58′–20°20′ N, 108°37′–117°50′ E) Province, China, to exclude the influence of other factors, such as growth conditions. The details of the 24 agarwood samples are shown in Table S1. In order to obtain a more comprehensive chemical characterization of agarwood samples, GC-MS was performed to obtain the volatile components of the ethanol extracts of agarwood samples and UPLC–Q–TOF–MS techniques were performed to obtain non-volatile components of the ethanol extracts of agarwood samples.

For GC-MS analysis, agarwood powder (0.2 g) was placed in 10 mL of 95.0% ethanol and then ultrasonicated for 40 min at room temperature. The extracts were transferred to a new tube after filtration, and the solution was removed by rotary evaporation. The evaporated sample was dissolved in 2 mL of absolute ethanol and filtered with a 0.22 μm filter for later use. For the UPLC/Q-TOF-MS analysis, agarwood powder (0.1 g) was placed in 10 mL of 95.0% ethanol and directly filtered through a 0.22 µm filter after ultrasonic extraction. For the in vitro activity assay, agarwood powder (5 g) was soaked in 500 mL of 95% ethanol for half an hour and then extracted with a slightly boiling reflux for 1 h. After filtering with 8 layers of gauze and a 0.22 µm filter membrane, the solution was removed by rotary evaporation. Finally, the evaporated samples were dissolved in DMSO to prepare 250 mg/mL for later use.

### 3.2. Microscopic Analysis and Content Determination of the Total Composition Groups

For microscopic analysis, agarwood samples were soaked in deionized water for 4 h until softened, and then cut crosswise into small transparent slices with a thin blade. Finally, the obtained transparent slices were placed on a slide and observed under a microscope at 100× magnification.

Determination of PEC content with reference to the method of Yang Jinling et al. [49]. The concentration of PEC in the extracted agarwood samples solution (same as the LC/MS analysis solution) was determined by using UV spectrophotometer according to the standard curve drawn.

For the determination of the total content of ethanol soluble compounds, we referred to the Ch. P method [6]. Agarwood sample powder was placed in 50 mL of 95% ethanol and refluxed for 1 h, then filtered, and 25 mL was placed in a constant-weight evaporation dish and finally weighed after complete removal of the solvent. The total content of ethanol soluble compounds was calculated according to the following formula:

$$E\%=\left[\left(m_{1}-m_{2}\right)\times 2/m_{0}\right]\times 100\%$$

where *m*_0_ and *m*_2_ represented the weight of the sample and the weight of the constant weight evaporation dish, respectively, and *m*_1_ represented the combined weight of the extract and the evaporation dish after solvent removal.

### 3.3. GC-MS Analysis Conditions

GC–MS analysis was performed in an Agilent 7890A GC system coupled with a 5975C MS detector (Agilent Technologies, Santa Clara, CA, USA). Separations were performed on an HP-5 MS column (30 m × 0.25 mm × 0.25 μm, Agilent). The GC settings were as follows: injection volume 1 µL, carrier gas helium 1 mL/min; injection mode, split 1:54; temperature programming set was initially to increase from 50 °C to 160 °C at 11 °C/min, then was increased to 170 °C at 2 °C/min for 10 min, raised at 10 °C/min to 270 °C, and increased to 300 °C at 3 °C/min for 5 min. The MS parameters were as follows: the mass spectra ranged from m/z 40 to 400, selective ion-monitoring (SIM) mode was used, and the ionization energy was 70 eV. The transfer line temperature and ion source temperature were both set to 250 °C.

### 3.4. UPLC/Q-TOF-MS Analysis Conditions

LC-MS analysis was performed using the Acquity UPLC system (Waters Corp, Milford, MA, USA) with an Acquity UPLC^®^ HSS T3 (2.1 × 100 mm, 1.8 µm, Waters) column coupled with a quadrupole time-of-flight tandem spectrometer (Waters Corp, Milford, MA, USA). The column temperature was 30 °C. The mobile phases were acetonitrile (A) and water with 0.1% formic acid (B) at a flow rate of 0.3 mL/min. The gradient elution program was as follows: 0–5 min, 15–30% A; 5–20 min, 30–40% A; 20–30 min, 40–50% A; and 30–35 min, 50–85% A. The sample injection volume was 1 µL. The electrospray ionization (ESI) source conditions were set as follows: capillary voltage, 2.5 KV; drying gas temperature, 400 °C; drying volume flow rate, 700 L/h; nebulizer pressure, 241.325 kPa; scanning detection was in positive and negative ion mode; collision energy was set to 10 V for MS mode and ramped from 20–40 V for MSE mode; mass range, 50–1200 m/z; and Lockspary was used for internal standard correction.

### 3.5. Data Preprocessing and Analysis

All GC/MS raw data were exported as CDF files, which were subsequently imported into XCMS-Online software (Version 3.7.1, the Scripps Center for Metabolomics, La Jolla, CA, USA) for peak alignment, retention time correction, and peak intensity calculation. Based on the grouping of samples in this study, the pairwise job was selected for metabolic analysis. In addition, “GC/Single Quad (matchedFilter)” was selected as the parameter set. The parameters were as follows: feature detection (method = matchedFilter, step = 0.25, fwhm = 3, steps = 2, snthresh = 10, mzdiff = 0.5, max = 100, prefilter intensity = 50,000), Rt correction (method = peakgroups, ignore sample class, bw = 10, mzwid = 0.25, minfrac = 1, minsamp = 1, missing = 5, extra = 1, smooth = loess, family = Gaussian), grouping (method = density, bw = 30, mzwid = 0.25, minfrac = 0.5, minsamp = 9), and Kruskal-Wallis test.

The raw data acquired by the UPLC/Q-TOF-MS system were converted to ABF format using the Reifycs Abf Converter and then imported to MS-DIAL version 4.70 (NSF-JST, Yokohama, Japan). MS-DIAL software was used for data preprocessing, including peak extraction, denoising, deconvolution, and peak alignment, and the workflow settings were set to default or reference XCMS processing parameters. After data preprocessing, we obtained a total of three data matrices that contained retention time, mass, and peak intensity for further multivariate statistical analysis.

### 3.6. Multivariate Analysis and the Identification of Optimized Features

Multivariate statistics analysis was performed with SIMCA software (version 14.1.0, Umetrics, Switzerland) based on candidate features with a significant univariate test as X-variables and the sample number as Y-variables. Full cross-validation was performed to estimate the ability of the models to make predictions. Significantly different features were identified and visualized by the 95% confidence interval for the projection value (VIP > 1) and S-line. The metabolite signature identifications were validated by (i) cross-checking their presence in the raw data, (ii) comparing their signatures to those present in NIST 17 (MS similarity > 800), and (iii) comparing the identifications with the existing identifications in the literature and reference standards.

### 3.7. Computer-Aided Screening

#### 3.7.1. Target Protein and Ligand Preparation

A total of 153 natural compounds in the two types of agarwood were collected from the literature for building a focused compound library. The compound library includes PECs and sesquiterpenoids (SESs), and the details of the compound library are presented in Table S2. SwissADME was used to evaluate and screen the ADME parameters (pharmacokinetic screening under GI absorption condition was high, and at least more than two of 5 different rule-based filters) of the small molecule compounds library. A total of 54 potential targets for treating related diseases with agarwood ethanol extract were initially identified from the literature, DrugBank database, and STITCH database, and the related diseases include diabetes, asthma, depression, Alzheimer’s disease, coronary artery disease, gastric ulcer, myocardial infarction and gastric tumor. The selection and acquisition of the target proteins with a high-resolution crystalline structure was based on the UniProt database and RCSB database, and either ligand molecules contained in the downloaded protein structure models or FDA-approved drugs were selected as positive drugs. The details of the genes and target proteins are presented in Table S3.

#### 3.7.2. Molecular Docking Simulation

AutoDock Vina (Trott and Olson, 2010) was used to predict the binding affinity and binding mode of compounds from the self-built compound libraries to candidate targets. The docked active-site pockets were obtained from the extension of the crystallographic ligand-binding site. The remaining program parameters used in the docking were the recommended defaults. In this study, the ligands with greater docking scores than those of positive drugs were considered to have strong binding affinity for potential targets. PYMOL version 2.4.0 (Schrödinger LLC, New York, NY, USA) was performed to visualize and analyze the binding mode of protein-ligand complexes.

#### 3.7.3. Network Analysis

The network analysis process was performed as follows. (1) The compound-target-pathway network structure was visualized by Cytoscape Version 3.9.0 (NIH, Bethesda, MD, USA). (2) The protein-protein interaction (PPI) network was established with STRING. (3) Gene Ontology (GO) and KEGG enrichment analyses were performed on the candidate targets by the DAVID database.

### 3.8. Cell Culture and Treatment

HEK293T cells was acquired from American Type Culture Collection. HEK293T cells were grown in DMEM (Invitrogen) supplemented with 10% FBS (Hyclone) at 37 °C in 5% CO2. To investigate whether the agarwood extract activates AMPK, 70–80% confluent 293T cells were treated with the agarwood extract alone for 1 h. All chemicals used in the treatment were dissolved in DMSO, and the final concentration of the extracts containing DMSO was 0.1%.

### 3.9. Western Blot Analysis

HEK293T cells were lysed by cell lysis solution containing protease inhibitor and phosphatase inhibitor at 4 degrees for 2 h. Cell lysates were then centrifuged to remove cell debris and were treated the sample with β-mercaptoethanol for 30 min, and boiled for 5 min to denature protein. Cell lysates were subjected to sodium dodecyl sulfate/polyacrylamide gel electrophoresis and incubated with anti-AMPK antibody (Cell Signaling) and anti-p-AMPK antibody (Cell Signaling) at 4 degrees overnight and followed by goat anti-rabbit horseradish-peroxidase-labeled secondary antibody (Santa Cruz) at room temperature for 1 h. The total amounts of pAMPK and total AMPK were measured as described [60,61], and their relative abundances were quantified using NIH ImageJ software (NIH, Bethesda, MD, USA). All experiments were performed at least three times and representative results were presented. Metformin and DMSO were used as positive controls and vehicle controls, respectively.

### 3.10. Statistical Analysis

Means and standard deviations were calculated for each parameter by accession. One-way analysis of variance (ANOVA) and mean comparisons were performed with the Statistical Analysis System (SAS) program. A result was considered statistically significant when *p* < 0.05.

## 4. Conclusions

In this study, multiple analytical methods were performed to reveal the intrinsic reasons for GKA as a high-quality agarwood. The total content of in ethanol soluble compounds as well as the total PECs content of GKA were significantly higher than those of normal agarwood. MS-based high-throughput analysis combined with multivariate statistical analysis revealed that GKA has higher concentrations of FTPECs (m/z 250–312) than normal agarwood. Molecular docking was performed to identify active ingredients and potential targets, which identified AMPK as the key target and FTPECs were found to have good binding ability to multiple targets. We also verified the activation ability of agarwood extract to activate AMPK in vitro, and the results showed that GKA exhibited a stronger activation ability than normal agarwood. Our results showed that GKA has high FTPECs with high activity, suggesting that GKA has the potential to be a medicinal plant resource for developing natural products with excellent biological activities.

## Figures and Tables

**Table 1:** Average content of the agarwood ethanol extract and the total PECs from 12 batches of GKA and 12 batches of normal agarwood. *p* values indicate significant differences between the tested extracts according to Tukey’s test.

Contents	Normal Agarwood	Grafted Kynam Agarwood	*p*-Value
Ethanol extracts (g/g)	0.223 ± 0.089	0.414 ± 0.080	0.000015235
Total PECs (mg/g)	102.197 ± 37.902	306.402 ± 65.450	2.96989E−08

## Data Availability

All data generated or analysed during this study are included in this published article and its Supplementary Materials Files.

## Supplementary Materials

The following supporting information can be downloaded at: https://www.mdpi.com/article/10.3390/molecules28041667/s1, Figure S1: An overview of the results of untargeted UPLC-Q-TOF-MS analysis of the metabolic profiles of all agarwood samples by MS-DIAL software. TIC overlay plot of raw data (**a**), overlay Plot of deconvolution results (**b**), the result of peak alignment (**c**); Figure S2: The chromatographic peak detection. Extracted ion chromatogram for 2-(2-phenythyl) chromone (**a**), visualization of the raw MS data for 2-(2-phenythyl) chromone, upper panel: chromatogram plotting the intensity values against the retention time, lower panel m/z against retention time plot. The individual data points are colored according to the intensity (**b**); Figure S3: Representative mass spectra of standards in the positive ESI mode; Table S1: The sample information; Table S2: Self-built compound library list, 153 natural compounds were collected from agarwood produced by A. sinensis; Table S3: Related targets for docking; Table S4: Compositional analysis of ethanol extracts of agarwood; Table S5. The preliminary identification of significantly different features screened from raw data obtained from GC-MS and UPLC-Q-TOF analysis of the two types of agarwood; Table S6: the top docking score over positive drugs was recorded in the docking results.

## Sample Availability

Samples of the compounds are available from the authors.

## Abbreviations

GKA, grafted Kynam agarwood; PECs, 2-(2-phenethyl) chromones; FTPECs, flindersia-type 2-(2-phenylethyl) chromones; THPECs, 5,6,7,8-tetrahydro-2-(2-phenylethyl) chromones; SESs, sesquiterpenoids; PCA, principal component analysis; OPLS-DA, orthogonal partial least squares discriminant analysis; AMPK, AMP-activated protein kinase; PECPS, diarylpentane-producing polyketide synthase.
