# Supplementary material for: Comprehensive Comparisons between Grafted Kynam Agarwood and Normal Agarwood on Traits, Composition, and In Vitro Activation of AMPK
Source: Molecules. 2023 Feb 9;28(4):1667. doi: 10.3390/molecules28041667 (PMC9961698; doi:10.3390/molecules28041667)
Supplement: Supplementary file 1 [file molecules-28-01667-s001.zip › molecules-2183153-supplementary.pdf]

SUPPLEMENTARY MATERIAL

Table S1. The sample information.

Num.	Type	Origin	Num.	Type	Origin
K1	Grafted Kynam-agarwood	Guangdong, China	OA1	Normal agarwood (Aquilaria sinensis)	HaiNan, China
K2	Grafted Kynam-agarwood	Guangdong, China	OA2	Normal agarwood (Aquilaria sinensis)	HaiNan, China
K3	Grafted Kynam-agarwood	Guangdong, China	OA3	Normal agarwood (Aquilaria sinensis)	Hong Kong, China
K4	Grafted Kynam-agarwood	Guangdong, China	OA4	Normal agarwood (Aquilaria sinensis)	Guangdong, China
K5	Grafted Kynam-agarwood	Guangdong, China	OA5	Normal agarwood (Aquilaria sinensis)	Guangdong, China
K6	Grafted Kynam-agarwood	Guangdong, China	OA6	Normal agarwood (Aquilaria sinensis)	HaiNan, China
K7	Grafted Kynam-agarwood	Guangdong, China	OA7	Normal agarwood (Aquilaria sinensis)	HaiNan, China
K8	Grafted Kynam-agarwood	Guangdong, China	OA8	Normal agarwood (Aquilaria sinensis)	HaiNan, China
K9	Grafted Kynam-agarwood	Guangdong, China	OA9	Normal agarwood (Aquilaria sinensis)	HaiNan, China
K10	Grafted Kynam-agarwood	Guangdong, China	OA10	Normal agarwood (Aquilaria sinensis)	Guangdong, China

K11	Grafted Kynam-agarwood	Guangdong, China	OA11	Normal agarwood (Aquilaria sinensis)	Guangdong, China
K12	Grafted Kynam-agarwood	HaiNan, China	OA12	Normal agarwood (Aquilaria sinensis)	Guangdong, China

Table S2. Self-built compound library list, 153 natural compounds were collected from agarwood produced by A. sinensis.

Num.	Name	CLASS	Num.	Name	CLASS	Num.	Name	CLASS
1	a-Agarofuran	Agarofurans	52	Ledol	Guaianes	103	6-Methoxy-2-[2-(4'-methoxyphenyl)ethyl]chromone	FTPECs
2	b-Agarofuran	Agarofurans	53	γ-Gurjunene	Guaianes	104	6,8-Dihydroxy-2-(2-phenylethyl)chromone	FTPECs
3	Dihydro-β-agarofuran	Agarofurans	54	Longifolene	Guaianes	105	6-Hydroxy-7-methoxy-2-(2-phenylethyl)chromone	FTPECs
4	Baimuxinol	Agarofurans	55	Guaiol	Guaianes	106	6-Methoxy-2-[2-(3-hydroxy-4-methoxyphenyl)ethyl]chromone	FTPECs
5	Dehydrobaimuxinol	Agarofurans	56	α-Cedrol	Guaianes	107	6-Hydroxy-2-[2-(3-hydroxy-4-methoxyphenyl)ethyl]chromone	FTPECs
6	4-Hydroxyl-baimuxinol	Agarofurans	57	11β-Hydroxy-13-isopropyl-dihydrodehydrocostus lactone	Guaianes	108	6-Hydroxy-7-methoxy-2-[2-(4-methoxyphenyl)ethyl]chromone	FTPECs
7	agarospirol	Agarospiranes	58	α-Patchoulene	Guaianes	109	6-Hydroxy-2-[2-(3,4-dimethoxyphenyl)ethyl]chromone	FTPECs
8	baimuxinal	Agarospiranes	59	Velleral	Guaianes	110	6,8-Dihydroxy-2-[2-(4-methoxyphenyl)ethyl]chromone	FTPECs
9	Baimuxinic acid (Bai Mu Xiang acid)	Agarospiranes	60	Isoaromadendrene epoxide	Guaianes	111	8-Chloro-6-hydroxy-2-[2-(3-methoxy-4-hydroxyphenyl)ethyl]chromone	FTPECs
10	Hinesol	Agarospiranes	61	Aromadendrene oxide-(1)	Guaianes	112	5-Methoxy-6-hydroxy-2-[2-(3-methoxy-4-hydroxyphenyl)ethyl]chromone	FTPECs

11	4-epi-15-Hydroxyacorenone	Agarospiranes	62	Aromadendrene oxide-(2)	Guaianes	113	7-Methoxy-2-[2-(4'-hydroxy-phenyl)ethyl]chromone	FTPECs
12	15-Hydroxyacorenone	Agarospiranes	63	Sinenofuranol	Guaianes	114	7-Hydroxy-2-[2-(4'-methoxyphenyl)ethyl]chromone	FTPECs
13	10-epi-γ-Eudesmol	Eudesmanes	64	3-Oxo-7-hydroxylholosericin A	Guaianes	115	5,6-Dihydroxy-2-[2-(3'-hydroxy-4'-methoxyphenyl)ethyl]chromone	FTPECs
14	(S)-4a-Methyl-2-(1-methylethyl)-3,4,4a,5,6,7-hexahydronaphthalene	Eudesmanes	65	Patchoulialcohol	Others	116	6-Hydroxy-5-methoxy-2-(2-phenyl-ethyl)chromone	FTPECs
15	Isolongifolene	Eudesmanes	66	(+)-8b-Hydroxy-longicamphenylone	Others	117	7-Methoxy-2-(2-phenylethyl)chromone	FTPECs
16	α-Eudesmol	Eudesmanes	67	Valerenic acid	Others	118	5-Hydroxy-2-(2-phenylethyl)chromone	FTPECs
17	α-Copaen-11-ol	Eudesmanes	68	Valerenal	Others	119	6,7-Dimethoxy-2-[2-(4-hydroxyphenyl)ethyl]chromone	FTPECs
18	β-Eudesmol	Eudesmanes	69	2,6-Dimethyl-10-methylene-12-oxatricyclo[7.3.1.0(1,6)]tridec-2-ene	Others	120	4',6-Dihydroxy-3',7-dimethoxy-2-(2-phenyl)ethyl chromone	FTPECs
19	β-Maaliene	Eudesmanes	70	β-Caryophyllene	Others	121	4'-Hydroxy-6-methoxy-2-(2-phenylethyl)chromone	FTPECs
20	α-Selinene	Eudesmanes	71	a-Humulene	Others	122	3',6-Dihydroxy-4'-methoxy-2-(2-phenylethyl)chromone	FTPECs

21	6-Isopropenyl-4,8a-dimethyl-1,2,3,5,6,7,8,8a-octahydro-naphthalen-2-ol	Eudesmanes	72	Humulene diepoxide A	Others	123	6,7-Dihydroxy-2-(2-phenylethyl)-5,6,7,8-tetrahydrochromone	THPECs
22	Selina-3,11-diene-12,15-dial	Eudesmanes	73	Kobusone	Others	124	8-Chloro-2-(2-phenylethyl)-5,6,7-trihydroxy-5,6,7,8-tetrahydrochromone	THPECs
23	Selina-3,11-dien-9,15-diol	Eudesmanes	74	Santalol	Others	125	8-Chloro-5,6,7-trihydroxy-2-(3-hydroxy-4-methoxyphenethyl)-5,6,7,8-tetrahydro-4H-chromen-4-one	THPECs
24	(7S,8R,10S)-(+)-8,12-Dihydroxy-selina-4,11-dien-14-al	Eudesmanes	75	Caryophyllenol-II	Others	126	5,6,7,8-Tetrahydroxy-2-(3-hydroxy-4-methoxyphenethyl)-5,6,7,8-tetrahydro-4H-chromen-4-one	THPECs
25	(7S,9S,10S)-(+)-9-Hydroxy-selina-4,11-dien-14-al	Eudesmanes	76	Baldrinal	Others	127	(5r,6s,7s,8r)-2-[2-(3'-Hydroxy-4'-Methoxyphenyl)Ethyl]-5,6,7,8-Tetrahydroxy-5,6,7,8-Tetrahydrochromone	THPECs
26	(5S,7S,9S,10S)-(-)-9-Hydroxy-selina-3,11-dien-14-al	Eudesmanes	77	α-Muurolene	Others	128	(5S,6R,7S)-5,6,7-Trihydroxy-2-(3-hydroxy-4-methoxyphenethyl)-5,6,7,8-tetrahydro- 4H-chromen-4-one	THPECs
27	(5S,7S,9S,10S)-(+)-9-Hydroxy-selina-3,11-dien-12-al	Eudesmanes	78	Elemol	Others	129	(5S,6R,7R)-5,6,7-Trihydroxy-2-(3-hydroxy-4-methoxyphenethyl)-5,6,7,8-tetrahydro-4H-chromen-4-one	THPECs
28	(5S,7S,9S,10S)-(+)-9-Hydroxy-eudesma-3,11(13)-dien-12-methylester	Eudesmanes	79	Cubenol	Others	130	Agarotetrol	THPECs

29	ent-4(15)-Eudesmen-11-ol-1-one	Eudesmanes	80	1,2,5,5,8a-Pentamethyl-1,2,3,5,6,7,8,8a-octahydronaphthalen-1-ol	Others	131	(5S,6S,7R,8S)-2-[2-(4-Methoxyphenyl)ethyl]-6,7,8-trihydroxy-5-methoxy-5,6,7,8-tetrahydrochromone	THPECs
30	Selina-4,11-diene-12,15-dial	Eudesmanes	81	1,5,9-Trimethyl-1,5,9-cyclododecatriene	Others	132	(5R,6R,7S,8R)-2-(2-Phenylethyl)-6,7,8-trihydroxy-5-methoxy-5,6,7,8-tetrahydrochromone	THPECs
31	(+)-9b-Hydroxyeudesma-4,11(13)-dien-12-al	Eudesmanes	82	2-(2-Phenylethyl)chromone	FTPECs	133	2-(2-Phenylethyl)-5,6,7,8-tetrahydroxy-5,6,7,8-tetrahydrochromone	THPECs
32	(+)-Eudesma-4,11(13)-dien-8a,9b-diol	Eudesmanes	83	6-Hydroxy-2-(2-phenylethyl)chromone	FTPECs	134	(5r,6s,7s,8r)-2-[2-(4'-Methoxyphenyl)Ethyl]-5,6,7,8-Tetrahydroxy-5,6,7,8-Tetrahydrochromone	THPECs
33	12,15-Dioxo-selina-4,11-dine	Eudesmanes	84	6-Methoxy-2-(2-phenylethyl)chromone	FTPECs	135	(5R,6R,7R,8S)-8-Chloro-5,6,7-trihydroxy-2-(4-methoxyphenethyl)-5,6,7,8- tetrahydrochromone	THPECs
34	(+)-8a-Hydroxyeudesma-3,11(13)-dien-14-al	Eudesmanes	85	6-Hydroxy-2-[2-(4-methoxyphenyl)ethyl]chromone	FTPECs	136	(5R,6S,7S)-5,6,7-Trihydroxy-2-(4-hydroxy-3-methoxyphenethyl)-5,6,7,8-tetrahydrochromone	THPECs
35	(+)-Eudesma-3,11(13)-dien-8α,9β-diol	Eudesmanes	86	6-Methoxy-2-[2-(3-methoxyphenyl)ethyl]chromone	FTPECs	137	5,6:7,8-Diepoxy-2-(2-phenylethyl)-5,6,7,8-tetrahydrochromone	DEPECs
36	(4R,5R,7S,9S,10S)-(-)-Eudesma-11(13)-en-4,9-diol	Eudesmanes	87	6,7-Dimethoxy-2-(2-phenylethyl)chromone	FTPECs	138	5,6:7,8-Diepoxy-2-[2-(4-methoxyphenyl)ethyl]-5,6,7,8-tetrahydrochromone	DEPECs
37	(+)-(4S,5R)-Dihydrokaranone	Eremophilanes	88	5,8-Dihydroxy-2-(2-phenylethyl)chromone	FTPECs	139	5,6-Epoxy-7β-hydroxy-8β-methoxy-2-(2-phenylethyl)chromone	DEPECs

38	Eremophila-9,11-dien-8-one (neopetasane)	Eremophilanes	89	5,8-Dihydroxy-2-[2-(4-methoxyphenyl)ethyl]chromone	FTPECs	140	rel-(1aR,2R,3R,7bS)-1a,2,3,7b-Tetrahydro-2,3-dihydroxy-5-[2-(4-methoxyphenyl) ethyl]-7H-oxireno[f][1]benzopyran-7-one	Dimeric PECs
39	(-)-(4R,5S,7R)-Jinkoh-eremol	Eremophilanes	90	6,7-Dimethoxy-2-[2-(4'-methoxyphenyl)ethyl]chromone	FTPECs	141	2-[2-(4-Glucosyloxy-3-methoxyphenyl)ethyl]chromone	Dimeric PECs
40	valerianol	Eremophilanes	91	6-Methoxy-2-[2-(3-methoxy-4-hydroxyphenyl)ethyl]chromone	FTPECs	142	Aquisinenone A	Dimeric PECs
41	Valencene	Eremophilanes	92	6-Hydroxy-2-[2-(3-methoxy-4-hydroxyphenyl)ethyl]chromone	FTPECs	143	(−)-4'-Methoxyaquisinenone A	Dimeric PECs
42	Aristolene	Eremophilanes	93	6-Hydroxy-2-(2-hydroxy-2-phenylethyl)chromone	FTPECs	144	Aquisinenone B	Dimeric PECs
43	Nootkatone	Eremophilanes	94	5-Hydroxy-6-methoxy-2-(2-phenylethyl)chromone	FTPECs	145	6'-Hydroxyaquisinenone B	Dimeric PECs
44	7α-H-9(10)-ene-11,12-epoxy-8-oxoeremophilane	Eremophilanes	95	6-Hydroxy-7-methoxy-2-[2-(3'-hydroxy-4'-methoxyphenyl)ethyl]chromone	FTPECs	146	6'-Hydroxy-4′,4'-dimethoxyaquisinenone B	Dimeric PECs
45	11-Hydroxy-valenc-1(10)-en-2-one	Eremophilanes	96	6,7-Dimethoxy-2-[2-(3'-hydroxy-4'-methoxyphenyl)ethyl]chromone	FTPECs	147	Aquisinenone C	Dimeric PECs

46	Ligudicin C	Eremophilanes	97	7-Hydroxy-6-methoxy-2-[2-(3'-hydroxy-4'-methoxy-phenyl)ethyl]chromone	FTPECs	148	(−)-Aquisinenone D	Dimeric PECs
47	(+)-9b,10b-Epoxyeremophila-11(13)-en	Eremophilanes	98	6,7-Dihydroxy-2-[2-(4'-methoxyphenyl)ethyl]chromone	FTPECs	149	4′-Demethoxyaquisinenone D	Dimeric PECs
48	(+)-11-Hydroxyvalenc-1(10),8-dien-2-one	Eremophilanes	99	6-Hydroxy-7-methoxy-2-[2-(4'-hydroxyphenyl)ethyl]chromone	FTPECs	150	Aquisinenone E	Dimeric PECs
49	epi-Ligulyl oxide	Guaianes	100	6,8-Dihydroxy-2-[2-(3'-hydroxy-4'-methoxyphenyl)ethyl]chromone	FTPECs	151	Aquisinenone F	Dimeric PECs
50	Sinenofuranol	Guaianes	101	6-Hydroxy-2-[2-(4'-hydroxy-3'-methoxyphenyl)ethenyl]chromone	FTPECs	152	Aquisinenone G	Dimeric PECs
51	Viridiflorol	Guaianes	102	2-[2-(4'-Methoxyphenyl)ethyl]chromone	FTPECs	153	4′-Methoxyaquisinenone G	Dimeric PECs

Table S3. Related targets for docking.

Num.	Gene	Target	PDB ID	Num.	Gene	Target	PDB ID
T1	HMGCR	HMGCR	1hw8	T28	GRIN1	GRIN1	5H8Q

T2	SLC5A2	SLC5A2	7VSI	T29	GRIN2B	GRIN2B	5EWJ
T3	ABCC8	ABCC8	6jb3	T30	ESR1	ESR1	7NFB
T4	PPARG	PPARG	6MS7	T31	GRIN2A	GRIN2A	5H8Q
T5	AGTR1	AGTR1	4ZUD	T32	ABL1	ABL1	4WA9
T6	DPP4	DPP4	4N8D	T33	AR	AR	1US0
T7	ADRB2	ADRB2	5X7D	T34	IGF1R	IGF1R	3D94
T8	NR3C1	NR3C1	4MDD	T35	MAPK14	MAPK14	3ROC
T9	HRH1	HRH1	3RZE	T36	MTOR	MTOR	5GPG
T10	CYSLTR1	CYSLTR1	6RZ4	T37	SIRT1	SIRT1	5BTR
T11	DRD2	DRD2	6CM4	T38	VDR	VDR	3A3Z
T12	HTR2A	HTR2A	7WC8	T39	AMY2A	AMY2A	4GQR
T13	SLC6A4	SLC6A4	5I6X	T40	BTK	BTK	3PIX
T14	ACHE	ACHE	4M0E	T41	EPHA2	EPHA2	5I9X
T15	CHRNA7	CHRNA7	5AFN	T42	EPHB4	EPHB4	6FNM
T16	PTGS1	PTGS1	4O1Z	T43	FKBP1A	FKBP1A	1BKF
T17	PTGS2	PTGS2	5IKR	T44	GABRG2	GABRG2	6X3T
T18	PTGER3	PTGER3	6M9T	T45	GHSR	GHSR	7NA7
T19	P2RY12	P2RY12	4NTJ	T46	LCK	LCK	2ZM1
T20	TNNC1	TNNC1	1WRK	T47	MGAM	MGAM	3CTT
T21	ACE	ACE	6H5W	T48	OXTR	OXTR	6TPK
T22	TYMS	TYMS	1HVY	T49	SI	SI	3LPP
T23	BCL2	BCL2	5MHQ	T50	SRMS	SRMS	4O75
T24	NR3C2	NR3C2	3VHV	T51	YES1	YES1	6G54
T25	NR1C2	PPARD	5U3Q	T52	ABL2	ABL2	3GVU
T26	NR1C3	PPARG	6MD4	T53	HCK	HCK	3VS3
T27	PPARA	PPARA	6KXX	T54	PRKAB1	PRKAB1	5ISO

Table S4. Compositional analysis of ethanol extracts of agarwood.

peak	Class	RT(min)		Formula	[M+H]- or [M+H]+	MS/MS Fragments	Substituent Group A Ring	Substituent Group B Ring	Proposed Compound	normal agarwood	Kynam-agarwood
1	THPECs	2.06	-	C18H20O8	363.1085	137 190 327	OH, OH, OH, OH	OH, OCH3	aquilarone a	√	
2	THPECs	2.44	-	C18H20O8	363.1234	137 190 327	OH, OH, OH, OH	OH, OCH3	aquilarone a(isomer 2)	√	
3	THPECs	2.71	-	C17H18O7	333.0924	317 299 271 243 164 193 137 107	OH, OH, OH, OH	OH, OCH3	aquilarone f	√	
4	THPECs	3.45	+	C17H18O6	319.1227	301 283 255 164 227 91	OH, OH, OH, OH	-	agarotetrol	√	
5	THPECs	3.56	+	C18H20O7	349.1325	331 313 285 121	OH, OH, OH, OH	OCH3	4-methoxyagarotetrol	√	
6	THPECs	3.885	+	C17H18O6	319.1188	301 255 283 227 164	OH, OH, OH, OH	-	iso-agarotetrol	√	
7	THPECs	4.000	+	C18H20O7	349.1290	331 313 285 121	OH, OH, OH, OH	OCH3	5,6,7,8-tetrahydroxy-2-(4-methoxyphenethyl)-5,6,7,8-tetrahydrochromone	√	
8	THPECs	4.07	+	C17H18O6	319.1212	301 283 255 227 164	OH, OH, OH, OH	-	aquilarone b	√	
9	THPECs	4.16	+	C18H20O7	349.1310	331 313 285 255 121	OH, OH, OH, OH	OCH3	5,6,7,8-tetrahydroxy-2-(4-methoxyphenethyl)-5,6,7,8-tetrahydrochromone(isomer 2)	√	
10	THPECs	4.23	+	C18H20O6	333.1337	303 285 255 121	OH, OH, OH, OCH3	-	5,6,7-trihydroxy-8-methoxy-5,6,7,8-tetrahydro-2-(2-phenylethyl)chromone	√	

11	THPE Cs	4.83	+	C17H18O 5	303.1237	285 267 239 176	OH, OH, OH	-	5,6,7-trihydroxy-5,6,7,8-tetrahydro-2-(2-phenylethyl)chromone	√
12	THPE Cs	5.54	+	C18H20O 6	333.1240	303 267 121	OH, OH, OH	OCH3	5,6,7-trihydroxy-2-(4-methoxyphenethyl)-5,6,7,8-tetrahydrochromone (isomer 2)	√
13	DEPE Cs	5.77	-	C18H16O 6	327.0873	137	-O-, -O-	OH, OCH3	5,6:7,8-Diedroxy-2-[2-(2-hydroxy-4-methoxy) phenylethyl]-5,6,7,8-tetrahydrochromone	√
14	THPE Cs	5.84	+	C17H18O 4	287.1286	269 251 239 178 160 121	OH, OH	-	6,7-dihydroxy-5,6,7,8-tetrahydro-2-(2-phenylethyl)chromone	√
15	FTPEC s	6.43		C18H16O 5	313.1089	161.0602	-	OH, OH, OCH3	2-[2-(3,5-hydroxy-4-methoxyl)phenylethyl] chromone	
16	DEPE Cs	6.42	-	C18H16O 6	327.0873	137 319 301 283 255	-O-, -O-	OH, OCH3	5,6:7,8-Diedroxy-2-[2-(3-hydroxy-4-methoxy) phenylethyl]-5,6,7,8-tetrahydrochromone	√
17	FTPEC s	6.573		C17H14O 4	283.0969	219 177 137 107	OH	OH	6-Hydroxy-2-[2-(3-hydroxyphenyl)ethyl] chromone	
18	THPE Cs	6.85	+	C18H19C lO6	367.0953	121 349	OH, OH, OH, Cl	OCH3	8-chloro-5,6,7-trihydroxy-2-(4-methoxyphenethyl)-5,6,7,8-tetrahydrochromene	√
19	THPE Cs	6.92	+	C17H17C 1O5	337.0858	319 283 265 192	OH, OH, OH, Cl	-	8-chloro-5,6,7-trihydroxy-2-(2-phenylethyl)-5,6,7,8-tetrahydrochromon	√
20	FTPEC s	7.26		C18H16O 5	313.1082	207 107 283 137	OH, OCH3	OH	6-hydroxy-7-methoxy-2-[2-(4-hydroxyphenyl)ethyl]chromone	
21	FTPEC s	7.43		C17H14O 4	283.0971	177	OH	OH	6-hydroxy-2-[2-(4-hydroxyphenyl)ethyl] chromone	
22	FTPEC s	7.47	+	C19H18O 6	343.1180	137 207 283	OH, OCH3	OH, OCH3	6-hydroxy-7-methoxy-2-(4-hydroxy-3-methoxyphenethyl) chromone	√

23	FTPECs	7.58	+	C19H18O6	343.1179	137 329 283	OH, OCH3	OH, OCH3	6-hydroxy-7-methoxy-2-(4-hydroxy-3-methoxyphenethyl) chromone(isomer 2)	√	
24	FTPECs	7.94	+	C19H18O6	343.1180	137	OH, OCH3	OH, OCH3	5-Hydroxy-6-methoxy-2-(4-hydroxy-3-methoxyphenethyl)chromone	√	
25	FTPECs	8.04	+	C17H14O4	283.0970	177 137 121	OH	OH	6-hydroxy-2-[2-(4-hydroxyphenyl)ethyl] chromone		
26	THPECs	8.21		C17H17C1O5	337.0839	319 283 265 121	OH, OH, OH, Cl	-	8-chloro-5,6,7-trihydroxy-2-(2-phenylethyl)-5,6,7,8-tetrahydrochromone (isomer 2)		
27	FTPECs	8.32		C18H16O5	313.1082	137 177	OH, OCH3	OH	6-Hydroxy-2-[2-(3-hydroxy-4-methoxyl)phenylethyl] chromone		
28	FTPECs	8.53		C17H14O4	283.0971	177	OH	OH	6-hydroxy-2-[2-(4-hydroxyphenyl)ethyl] chromone (isomer 2)		
29	FTPECs	8.85	+	C18H16O5	313.1077	137 283	OH	OH, OCH3	6-Hydroxy-2-[2-(3-methoxyl-4-hydroxy)phenylethyl]-chromone	√	√
30	FTPECs	9.06		C18H16O5	313.1082	121	OH, OH	OCH3	6,8-Dihydroxy-2-[2-(4-methoxy)phenylethyl]chromone		
31	FTPECs	9.28		C17H14O4	283.0989	192 91 164 337 122	OH, OH	-	6,8-Dihydroxy-2-(2-phenylethyl)chromone		
32	FTPECs	9.62	+	C19H18O4	311.0918	160 279 121 251	-	OCH3, OCH3	2-[2-(3,4-Dimethoxyphenyl)ethyl] chromone	√	√
33	FTPECs	9.99		C17H14O4	283.0970	177	OH	OH	6-hydroxy-2-[2-(4-hydroxyphenyl)ethyl] chromone (isomer 3)		
34	FTPECs	10.01		C19H18O5	327.1234	221 283 301 177 107 205 137	OCH3, OCH3	OH	qinanone g		

35	FTPECs	10.27		$C_{17}H_{14}O_3$	267.1043	161	-	OH	2-[2-(3-Hydroxyphenyl)ethyl]chromone		
36	FTPECs	10.274		$C_{20}H_{20}O_6$	357.1335	161 137	OCH3, OCH3	OH, OCH3	6,7-dimethoxy-2-[2-(3-hydroxy-4-methoxyphenyl)ethyl]chromone		
37	FTPECs	10.55		$C_{18}H_{16}O_5$	313.108	121	OH, OH	OCH3	6,7-dihydroxy-2-[2-(4-methoxyphenyl)ethyl]chromone		
38	FTPECs	10.85		$C_{17}H_{14}O_4$	283.0978	192 153 91 126	OH, OH	-	6,8-dihydroxy-2-(2-phenylethyl)chromone		
39	FTPECs	11.03		$C_{20}H_{20}O_6$	357.1338	137	OCH3, OCH3	OH, OCH3	6,8-dimethoxy-2-[2-(3-hydroxy-4-methoxyphenyl)ethyl]chromone		
40	FTPECs	11.64	+	$C_{17}H_{14}O_3$	267.1021	161	-	OH	2-[2-(4-Hydroxy)phenylethyl]chromone	√	√
41	FTPECs	12.02	+	$C_{18}H_{16}O_4$	297.1127	137	-	OH, OCH3	2-[2-(3-Methoxy-4-hydroxy)phenylethyl]chromone		√
42	FTPECs	12.31	-	$C_{17}H_{14}O_3$	265.0863	107 160 121 137	-	OH	2-[2-(4-Hydroxy)phenylethyl]chromone(isomer 2)	√	√
43	BI	12.66		$C_{34}H_{30}O_9$	583.1968	283			AH15		
44	FTPECs	13.25	+	$C_{18}H_{16}O_4$	297.1125	121 137	OH	OCH3	6-hydroxy-2-[2-(4-methoxyphenyl)ethyl] chromone	√	
45	FTPECs	13.72	+	$C_{19}H_{18}O_5$	327.1238	121 191	OH, OCH3	OCH3	6-Methoxy-7-hydroxy-2-[2-(4-methoxyphenyl)ethyl] chromone	√	
46	FTPECs	14.11	+	$C_{19}H_{18}O_5$	327.1243	206 137 191 121	OCH3	OH, OCH3	6-Methoxy-2-[2-(3-methoxy-4-hydroxy)phenylethyl]-chromone		√
47	FTPECs	14.39	+	$C_{18}H_{16}O_4$	297.1145	283 206 191 137 121	OH, OCH3	-	6-Hydroxy-7-methoxy-2-(2-phenylethyl)chromone	√	

48	FTPECs	14.61	+	C17H14O3	267.1087	161 107 121 173	-	OH	2-[2-(2-Hydroxyphenyl)ethyl]chromone		√
49	BI	14.91	-	C34H30O9	581.1809	281			AH15(isomer 2)	√	
50	FTPECs	14.93	-	C18H16O4	295.0970	159	-	OH, OCH3	2-[2-(3-Hydroxy-4-methoxyphenyl)ethyl]chromone		√
51	FTPECs	15.08	+	C19H18O5	327.1229	137	OCH3	OH, OCH3	6-Methoxy-2-[2-(3-hydroxy-4-methoxyphenyl)ethyl]-chromone		√
52	FTPECs	15.60	+	C18H16O4	297.1143	121	OH	OCH3	6-Hydroxy-2-[2-(4-methoxyphenyl)ethyl]chromone(isomer 2)	√	√
53	FTPECs	16.09	+	C17H14O3	267.1076	177 137 147 91 110	OH	-	6-Hydroxy-2-(2-phenylethyl)chromone	√	√
54	BI	16.42	+	C35H32O9	597.2119	297.1124			AH12	√	
55	FTPECs	16.72	+	C19H18O4	311.1323	151 107	-	OCH3, OCH3	2-[2-(3,4-Dimethoxyphenyl)ethyl]chromone		√
56	BI	16.72	+	C34H30O9	583.1969				AH15(isomer 3)	√	
57	FTPECs	19.02	+	C20H20O5	341.1430	121	OCH3, OCH3	OCH3	6,7-Dimethoxy-2-[2-(4-methoxyphenyl)ethyl]chromone	√	
58	FTPECs	19.48	+	C19H18O4	311.1345	220	OCH3, OCH3	-	6,7-Dimethoxy-2-(2-phenylethyl)chromone	√	
59	FTPECs	19.64	+	C19H18O4	311.1348	220	OCH3, OCH3	-	6,7-Dimethoxy-2-(2-phenylethyl)chromone(isomer 2)	√	
60	FTPECs	20.54	+	C17H14O4	283.0969	255 192 153 121	OH	OH	6-hydroxy-2-[2-(4-hydroxyphenyl)ethyl] chromone	√	

61	BI	21.50	+	C33H38O10	591.2029	221 203			sesquiterpene-2-(2-phenylethyl) chromone derivative		√
62	BI	21.88	-	C34H28O8	565.1863	267			AH21		√
63	FTPECs	22.44	+	C18H16O3	281.1177	121	-	OCH3	2-[2-(4-methoxyphenyl)ethyl]chromone	√	√
64	BI	22.69	+	C34H30O8	567.2018	283 549 267 239 121			AH13	√	
65	FTPECs	23.02	+	C17H14O2	251.1111	160 121 91	-	-	2-(2-phenylethyl)chromone	√	√
66	BI	23.55	+	C34H28O8	565.1862	281 190 151 121			AH21(isomer 2)	√	√
67	FTPECs	23.69	+	C18H16O3	281.1177	190 151 121 91	OCH3	-	6-methoxy-2-(2-phenylethyl)chromone	√	√
68	EPECs	24.51	+	C17H16O5	301.0633	283 210 121 137 171 91	OH, OH, -O-	-	5,6-Edroxy-7,8-dihydroxy-2-(2-phenylethyl)-5,6,7,8-tetrahydrochromone	√	
69	FTPECs	24.99	+	C19H18O4	311.1306	190 121 151 147 91	OCH3	OCH3	6-methoxy-2-[2-(4-methoxyphenyl)ethyl]chromone		√
70	FTPECs	25.53	+	C18H16O3	281.1225	190 151 147 124 91	OCH3	-	6-Methoxy-2-(2-phenylethyl)chromone(isomer 2)	√	√
71	FTPECs	27.48	+	C19H18O5	327.1229	283 121 206 163 91	OH, OCH3	OCH3	7-hydroxy-6-methoxy-2-(4-methoxyphenethyl)chromone	√	
72	FTPECs	28.13	+	C18H16O4	297.1130	206 163 121 91	OH, OCH3	-	5-hydroxy-6-methoxy-2-(2-phenylethyl)chromone	√	
73	BI	28.53	+	C36H30O8	591.2026	219 251			bi-2-(2-phenylethyl)chromone		√

73	BI	31.88	+	C35H30O8	579.2019	283 121 458	methoxy AH21	√	
74	BI	32.22	+	C34H28O7	549.1949	458 283 267 159 137	dehydroxy AH21	√	
75	BI	32.85	+	C34H28O7	549.1914	283 121 313	dehydroxy AH21	√	
76	TRI	32.91	+	C51H46O14	883.2736	723 429 335 297	tri-2-(2-phenylethyl)chromone		√

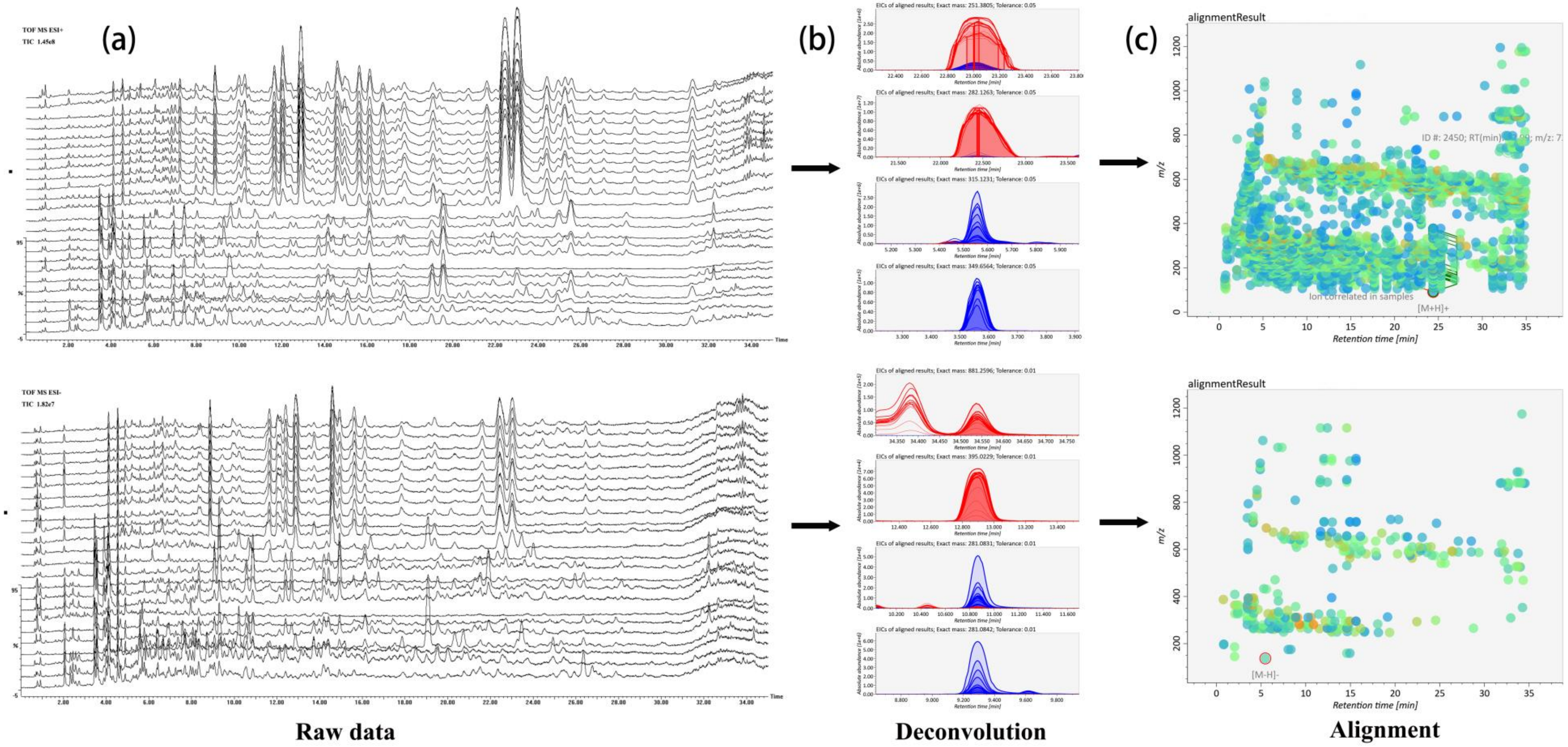

Figure S1. An overview of the results of untargeted UPLC-Q-TOF-MS analysis of the metabolic profiles of all agarwood samples by MS-DIAL software. TIC overlay plot of raw data (**a**), overlay Plot of deconvolution results (**b**), the result of peak alignment (**c**).

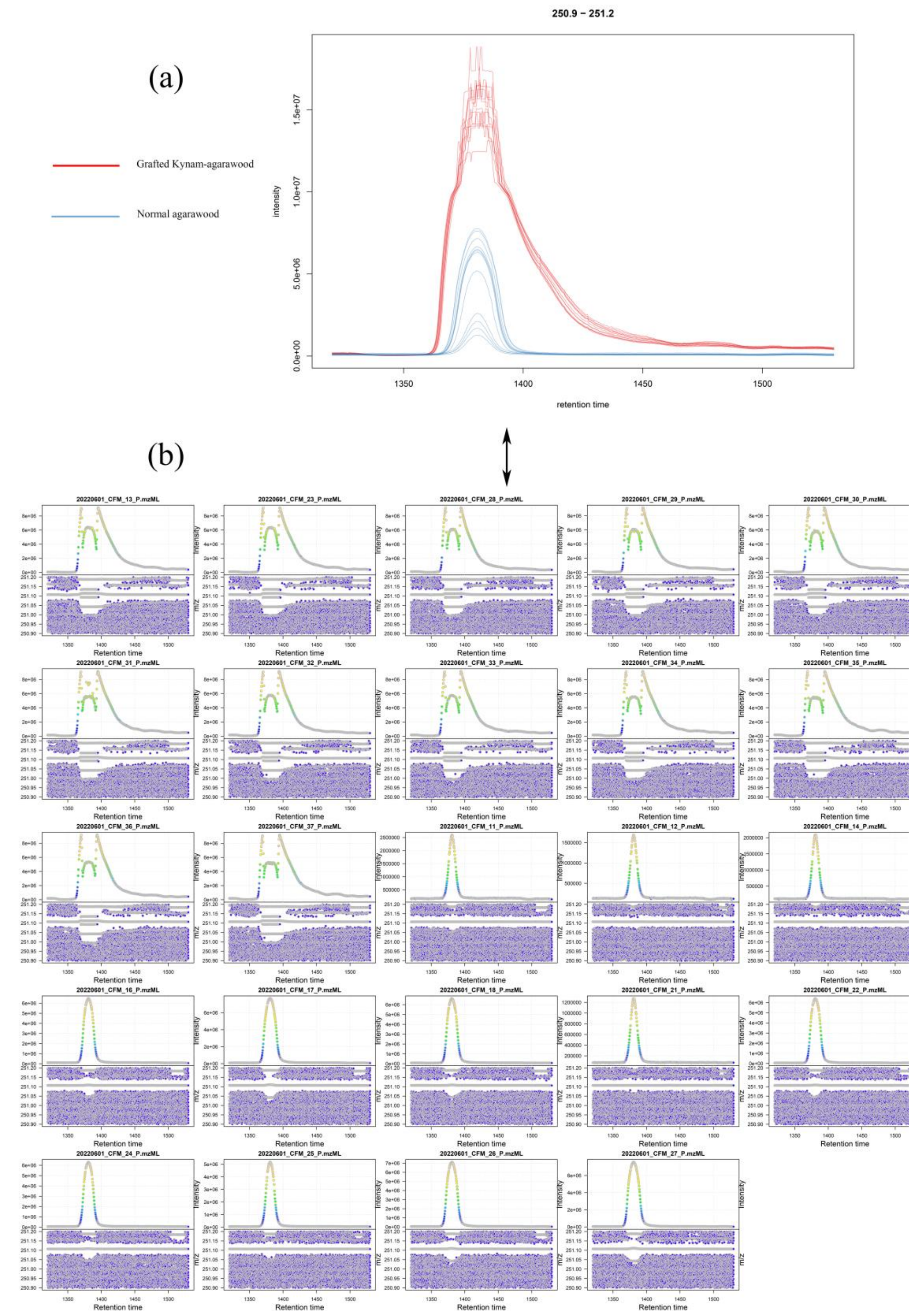

Figure S2. The chromatographic peak detection. Extracted ion chromatogram for 2-(2-phenythyl) chromone (**a**), visualization of the raw MS data for 2-(2-phenythyl) chromone, upper panel: chromatogram plotting the intensity

values against the retention time, lower panel m/z against retention time plot.

The individual data points are colored according to the intensity (**b**).

Table S5. The preliminary identification of significantly different features screened from raw data obtained from GC–MS and UPLC-Q-TOF analysis of the two types of agarwood.

Featureidx[a]	Up/DownTrend[b]	Class	Rt (min)	Observed features	The preliminary identification of markers	MW	Quantifedion[c]	P-Value[d]
**GC EI**								
262 235 221 446	DOWN	FTPECs	43.87	341.150 121.050 122.050 340.144	6,7-dimethoxy-2-[2-(4-methoxyphenyl)ethyl] chromone (MS)	340	121.050	7.400E-05
448 436	DOWN	FTPECs	43.50	328.116 137.056	6,8-dihydroxy-2-[2-(3-hydroxy-4-methoxyphenyl)ethyl] chromone (MS)	328	328.116	9.069E-03
355	DOWN	FTPECs	40.92	121.050	6,7-dihydroxy-2-[2-(4-methoxyphenyl) ethyl] chromone (MS)	312	121.050	1.847E-03
337 426	DOWN	FTPECs	40.62	121.016 312.100	6,8-dihydroxy-2-[2-(4-methoxyphenyl) ethyl] chromone (MS)	312	121.016	1.241E-03
434	DOWN	FTPECs	40.16	310.128	6-Methoxy-2-[2-(3-methoxyphenyl) ethyl] chromone (MS)	310	310.128	6.934E-03
145 162 126 175 193 104 140 151	UP	FTPECs	39.89	161.050 131.050 160.050 159.050 297.068 132.040 296.100 135.002	2-[2-(3-hydroxy-4-methoxyphenyl) ethyl] chromone (MS)	296	161.050	5.848E-06
202	UP	FTPECs	39.67	151.050	6-Methoxy-2-[2-(4-methoxyphenyl) eyhyl] chromone (R.match 824)	310	151.050	2.869E-05

327 404 74	UP	FTPECs	37.92	120.978 120.041 121.900	2-[2-(4-Methoxyphenyl) ethyl] chromone (R.match 888)	280	120.978	1.016E-03
90 135	UP	FTPECs	37.63	279.148 278.15	6-Methoxy-2-(2-phenylethyl) chromone (R.match 951)	280	279.148	1.866E-07
1 37	UP	FTPECs	34.59	91.940 250.023	2-(2-phenylethyl) chromone (R.match 964)	250	91.940	8.171E-13
42	UP	SESs	34.49	232.002	Guaia-1(10),11-dien-15,2-olide (MS)	232	232.002	5.449E-10
231 80 84 197	UP	FTPECs	33.92	266.029 267.994 265.050 268.975	6-Hydroxy-2-(2-phenylethyl) chromone (MS)	266	266.029	6.110E-05
83	UP	FTPECs	33.79	160.050	2-[2-(2-hydroxyphenyl) ethyl] chromone (MS)	266	160.050	9.159E-08
258	UP	Others	28.70	256.181	n-Hexadecanoic acid (R.match 814)	256	256.18	1.474E-04
220 223 222 233 253	UP	SESs	26.02	201.000 183.050 216.003 217.000 146.100	6-(1-Hydroxymethylvinyl)-4,8a-dimethyl-3,5,6,7,8,8a-hexahydro-1H-naphthalen-2-one (R.match 835)	234	183.050	4.865E-05
171 229	UP	SESs	24.55	216.002 201.000	Nootkaton-11,12-epoxide (R.match 804)	234	216.002	1.166E-05
70 98 109 192 190 204	UP	SESs	23.71	220.523 179.100 195.052 194.097 139.097 126.050	Isoaromadendrene epoxide (R.match 845)	220	220.523	8.975E-09
114	UP	SESs	17.21	152.102	(-)-longifolol (R.match 864)	222	152.102	1.569E-06
**UPLC ESI+**								
1110 1023	Down	THPECs	1.626	335.1128 317.1021	aquilarone f	334	335.1128	8.920E-07
1111	Down	THPECs	1.951	335.1129	aquilarone f (isomer)	334	335.1129	1.908E-06
1172	Down	THPECs	2.056	365.1242	5,6,7,8-Tetrahydroxy-2-(3-hydroxy-4-methoxyphenethyl)-5,6,7,8-tetrahydro-4H-chromen-4-one (aquilarone a)	364	365.124	1.008E-05
1039 950 839 1381 710 1871 1122 1356 1865 1910 1879 1350 1908	Down	THPECs	3.458	319.1237 301.1111 283.0969 505.1344 255.1021 659.2086 341.0996 497.1480 656.2026 690.1386 661.1872 489.1588 688.1437	agarotetrol	318	319.1237	1.049E-16

1092 995 1150 1180 1184 1285 442 1931	Down	THPECs	3.56	331.1191 313.1076 349.1336 368.1035 371.1104 448.0453 214.5557 719.2299	4'-methoxyagarotetrol	348	349.134	3.044E-08
1037 1123 947	Down	THPECs	3.896	319.1212 341.0998 301.1090	iso-agarotetrol	318	319.1212	5.151E-12
1148	Down	THPECs	3.999	349.1298	(5S,6R,7R)-5,6,7-Trihydroxy-2-[2-(3-hydroxy-4-methoxyphenyl) ethyl]-5,6,7,8-tetrahydrochromone	348	349.1298	7.861E-07
1038	Down	THPECs	4.071	319.1226	aquilarone b	318	319.1226	2.140E-07
1149	Down	THPECs	4.156	349.1314	(5S,6R,7R)-5,6,7-Trihydroxy-2-[2-(3-hydroxy-4-methoxyphenyl) ethyl]-5,6,7,8-tetrahydrochromone	348	349.1314	3.184E-06
377 501 168	UP	SESs	4.476	201.1645 219.1749 159.1174	sesquiterpene derivatives C15H22O	218	219.1749	4.817E-07
1138	Down	EPECs	4.66	347.1146	5,6-Edroxy-7,8-dihydroxy-2-[2-(3-hydroxy-4-methoxy)-phenylethyl]-5,6,7,8-tetrahydrochromone	346	347.1146	9.392E-10
494	UP	SESs	5.225	219.1747	sesquiterpene derivatives C15H22O	218	219.1747	1.383E-08
601	UP	SESs	5.26	235.1699	sesquiterpene derivatives C15H22O2	234	235.1699	6.191E-08
969 780 1671 875 630	Up	THPECs	5.396	309.1104 269.1180 287.1284 239.1073	6,7-dihydroxy-5,6,7,8-tetrahydro-2-(2-phenylethyl) chromone	286	287.1284	1.427E-11
588 588 303 408 473 342	Up	SESs	5.764	235.1697 189.1642 205.1591 217.1593 199.1486	sesquiterpene derivatives C15H22O2	234	235.1697	1.163E-11
182	Up	FTPECs	5.803	161.0604	5,8-Dihydroxy-2-(2-phenylethyl) chromone	282	161.0604	9.287E-14
836 987	Up	FTPECs	6.572	283.0968 313.1073	6-Hydroxy-2-[2-(3-hydroxy-4-methoxyphenyl) ethyl] chromone	312	313.1073	3.022E-07
482 610	Up	SESs	6.827	217.1595 235.1702	sesquiterpene derivatives C15H22O2	234	235.1702	5.888E-11
1005	Up	FTPECs	7.128	313.1079	6-Hydroxy-2-[2-(3-methoxy-4-hydroxyphenyl) ethyl] chromone	312	313.1079	3.422E-07
854	Up	FTPECs	8.411	283.0978	6,8-Dihydroxy-2-(2-phenylethyl) chromone	282	283.0978	9.779E-10

1010 84 1797 1106	Up	FTPECs	8.863	313.1143 137.0607 625.2079 335.0897	6-Hydroxy-2-[2-(3-hydroxy-4-methoxyl) phenylethyl]-chromone	312	313.1143	2.561E-08
1008	Down	FTPECs	9.053	313.1108	6,8-Dihydroxy-2-[2-(4-methoxyphenyl) ethyl] chromone	312	313.1108	8.326E-05
613 485	Up	SESs	9.991	235.1722 217.1596	sesquiterpene derivatives C15H22O2	234	235.1722	3.829E-10
916	Up	FTPECs	10.182	297.1126	2-[2-(3-hydroxy-4-Methox) phenylethyl] chromone	296	297.1126	3.380E-09
189 769	Up	FTPECs	10.264	161.0639 267.1049	2-[2-(4-Hydroxyphenyl) ethyl] chromone	266	267.1049	3.708E-20
1007	Down	FTPECs	10.558	313.1092	6,7-Dihydroxy-2-[2-(4-methoxyphenyl) ethyl] chromone	312	313.1092	3.847E-10
857	Down	FTPECs	10.861	283.1027	5,8-dihydroxy-2-(2-phenylethyl) chromone	282	283.1027	8.505E-15
597	Up	SESs	11.043	235.1698	sesquiterpene derivatives C15H22O2	234	235.1698	3.780E-07
750 772 186	Up	FTPECs	11.642	265.0895 267.1086 161.0607	qinanone d	266	267.1086	3.972E-23
929 187 85 940 1034 1850	Up	FTPECs	12.036	297.1195 161.0609 137.0608 300.3788 319.0946 646.1289	qinanone a	296	297.1195	1.043E-19
770	Up	FTPECs	12.388	267.1036	qinanone d (Isomer)	266	267.1036	1.041E-13
564	Up	SESs	12.706	233.1541	sesquiterpene derivatives C15H20O2	232	233.1541	1.587E-08
1036 931 86 939 930 1849 1735 913	Up	FTPECs	12.887	319.0948 298.1222 137.0609 300.3784 297.2998 646.1287 612.1924 297.1125	2-[2-(3-Methoxy-4-hydroxyphenyl) ethyl] chromone (qinanone b)	296	297.2998	7.065E-16
599	Up	SESs	13.253	235.1699	sesquiterpene derivatives C15H22O2	234	235.1699	1.383E-12
571	Up	SESs	13.264	233.1542	sesquiterpene derivatives C15H20O2	232	233.1542	6.394E-10
366 498	Up	SESs	13.404	201.1643 219.1748	sesquiterpene derivatives C15H22O	218	219.1748	6.967E-11
773 183	Up	FTPECs	14.589	267.1088 161.0604	qinanone d	266	267.1088	8.157E-31
926 82 180	Up	FTPECs	14.936	297.1159 137.0604 161.0603	qinanone c	296	297.1159	6.249E+06
570	Up	SESs	15.459	233.1542	sesquiterpene derivatives C15H20O2	232	233.1542	2.465E-09
600	Up	SESs	16.05	235.1699	sesquiterpene derivatives C15H22O2	234	235.1699	7.119E-09

980 143 1096 1773	Up	FTPECs	16.724	311.1335	6-Methoxy-2-[2-(4-methoxy) phenylethyl] chromone	310	311.1335	9.138E-21
1125	Down	FTPECs	19.018	341.1432	6,7-dimethoxy-2-[2-(4-methoxyphenyl) ethyl] chromone	340	341.1432	9.231E-11
612 496 481 350	Up	SESs	19.025	235.1707 219.1748 217.1594 199.1488	sesquiterpene derivatives C15H22O2	234	235.1708	4.580E-15
608	Up	SESs	19.381	235.1700	sesquiterpene derivatives C15H22O2	234	235.1700	3.952E-08
981	Down	FTPECs	19.567	311.1352	6,7-dimethoxy-2-(2-phenylethyl) chromone	310	311.1352	3.900E-09
519 392	Up	SESs	21.597	221.1905 203.1801	sesquiterpenes C15H24O	220	221.1905	3.958E-13
45 1286 1258 1608 1587 835 1634 957	Up	FTPECs	22.454	121.0664 448.1335 440.1467 588.1888 580.2022 281.1244 591.2006 303.1002	2-[2-(4-Methoxyphenyl) ethyl] chromone	280	281.1244	6.772E-19
697 684 178 696 1225 1414	Up	FTPECs	23.026	252.1144 251.1111 160.0526 251.2796 403.1173 520.1810	2-(2-phenylethyl) chromone	250	251.2796	1.319E-19
832	Up	FTPECs	23.307	281.1175	6-Methoxy-2-(2-phenylethyl) chromone	280	281.1175	1.802E-13
715 584 461 295	Up	SESs	24.402	255.1363 233.1548 215.1444 187.1489	sesquiterpene derivatives C15H20O2	232	233.1548	2.624E-10
582 460 713	Up	SESs	25.247	233.1545 215.1442 255.1358	sesquiterpene derivatives C15H20O2	232	233.1545	2.316E-10
322 512	Up	SESs	31.243	191.1800 219.1778	sesquiterpene derivatives C15H22O	218	191.18	1.317E-09
511	Up	SESs	32.288	219.1768	sesquiterpene derivatives C15H22O	218	219.1768	2.097E-11
**UPLC ESI-**								
120 136 102	Down	THPECs	2.06	363.1079 409.1132 327.0871	5,6,7,8-Tetrahydroxy-2-(3-hydroxy-4-methoxyphenethyl)-5,6,7,8-tetrahydro-4H-chromen-4-one (aquilarone a)	364	363.108	1.325E-05
122	Down	THPECs	2.44	363.1080	aquilarone e	364	363.108	2.095E-05
125 126 98 81	Down	THPECs	3.46	363.1090 363.2992 317.1032 299.0931	aquilarone f	364	363.109	8.963E-08

55 46 139	Up	SESs	5.27	279.1229 267.1594 433.1500	2-(4-Butoxybenzyl) succinic acid	280	279.123	1.896E-07
73	Up	FTPECs	12.18	295.0978	qinanone a	296	295.098	5.813E-11
16 74 15	Up	FTPECs	12.88	251.1650 295.0978 249.1498	2-[2-(3-Methoxy-4-hydroxyphenyl) ethyl] chromone (qinanone b)	296	295.098	1.490E-10
43 34 2	Up	FTPECs	14.59	265.25 265.087 159.045	qinanone d	266	265.087	4.597E-17
72 243 284 176 3	Up	FTPECs	14.94	295.0977 716.0662 976.1937 591.2019 159.0452	qinanone c	296	295.098	3.973E-10
71	Up	FTPECs	15.62	295.0975	6-Hydroxy-2-[2-(4-methoxyphenyl) ethyl] chromone	296	295.098	1.648E-09
185	Up	Dimers	23.02	605.1813	sesquiterpene-2- (2-phenylethyl) chromone derivative	606	605.181	4.706E-06
264	Up	Trimers	33.66	883.2744	tri-2-(2-phenylethyl) chromone	883	883.274	6.710E-08

[a] The numbers are the code for the MS features and correspond to those in Figure 3.

[b] Up/down trend indicates the difference in the expression levels of metabolites in grafting Kynam-Agarwood and Agarwood, up indicates a higher expression level in grafting Kynam-Agarwood, and DOWN is the opposite.

[c] The ion features with larger m/z values and stronger peak intensities in the same metabolite were selected as quantified ions.

[d] The p value is calculated using intensities of quantified ions by Welch's t test.

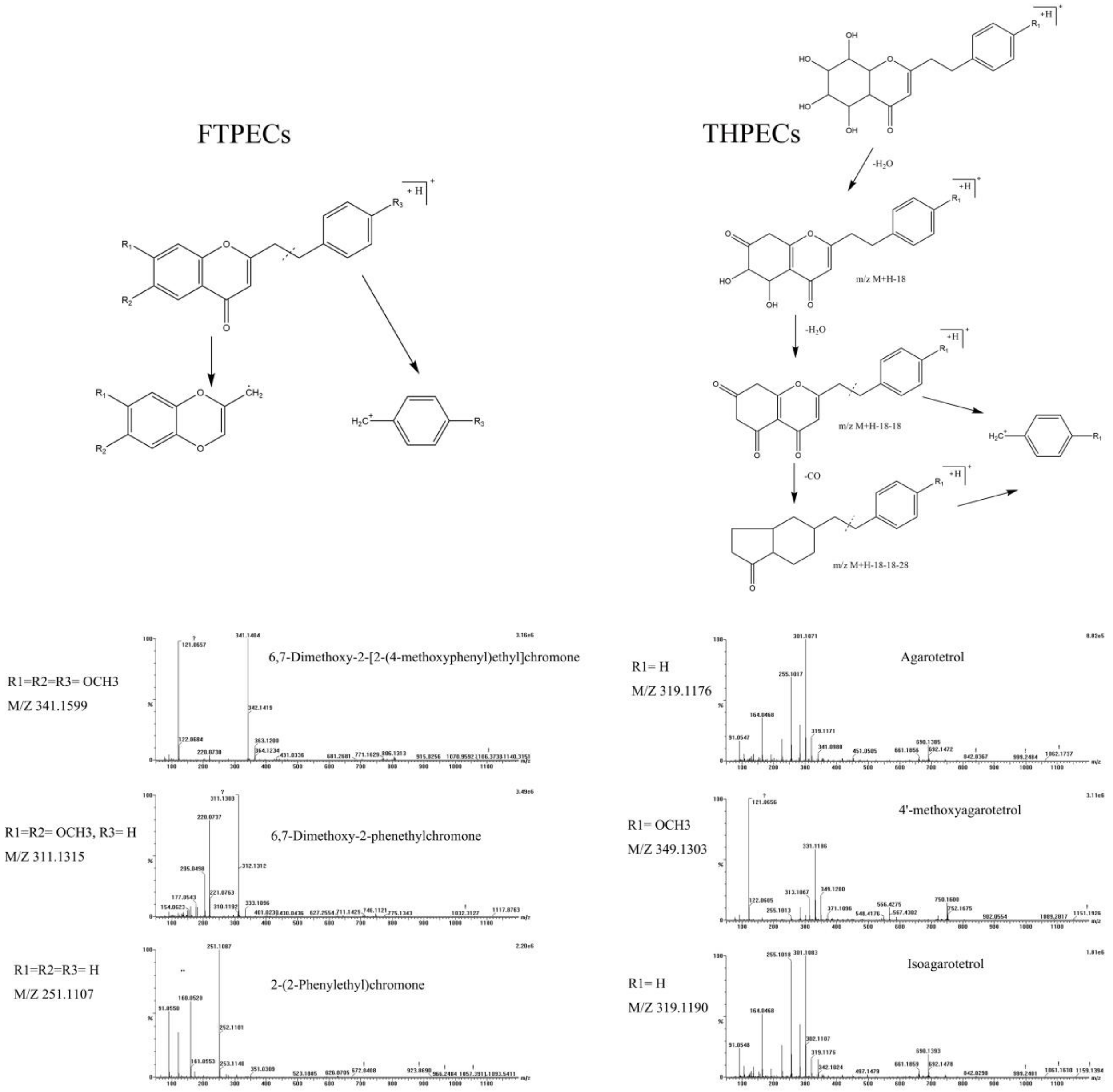
FTPECs
THPECs
-H2O
m/z M+H-18
-H2O
m/z M+H-18-18
-CO
m/z M+H-18-18-28
R1=R2=R3= OCH3
M/Z 341.1599
6,7-Dimethoxy-2-[2-(4-methoxyphenyl)ethyl]chromone
R1=R2= OCH3, R3= H
M/Z 311.1315
6,7-Dimethoxy-2-phenethylchromone
R1=R2=R3= H
M/Z 251.1107
2-(2-Phenylethyl)chromone
R1= H
M/Z 319.1176
Agarotetrol
R1= OCH3
M/Z 349.1303
4'-methoxyagarotetrol
R1= H
M/Z 319.1190
Isoagarotetrol

Figure S3. Representative mass spectra of standards in the positive ESI mode.

Table S6. the top docking score over positive drugs was recorded in the docking results.

ligand_NUM	receptor_PDB ID	Scores	ligand_NUM	receptor_PDB ID	Scores	ligand_NUM	receptor_PDB ID	Scores	ligand_NUM	receptor_PDB ID	Scores	ligand_NUM	receptor_PDB ID	Scores
151	1hvy	-11.6	112	3lpp	-7.2	86	4o1z	-9	61	6g54	-7.7	119	6ms7	-8.7
147	1hvy	-11.5	126	3lpp	-7.2	151	4o75	-10.2	83	6g54	-7.7	120	6ms7	-8.7
142	1hvy	-10.9	129	3lpp	-7.2	149	4o75	-10.1	96	6g54	-7.7	126	6ms7	-8.7
143	1hvy	-10.7	85	3lpp	-7.2	144	4o75	-10	150	6h5w	-11.5	132	6ms7	-8.7
150	1hvy	-10.4	86	3lpp	-7.2	150	4o75	-9.9	144	6h5w	-11.4	140	6ms7	-8.7
152	1hvy	-10.3	89	3lpp	-7.2	142	4o75	-9.8	147	6h5w	-11.3	85	6ms7	-8.7
145	1hvy	-10	103	3lpp	-7.1	143	4o75	-9.7	143	6h5w	-11.1	88	6ms7	-8.7
136	1hvy	-9.9	106	3lpp	-7.1	145	4o75	-9.6	151	6h5w	-11.1	89	6ms7	-8.7
146	1hvy	-9.9	125	3lpp	-7.1	152	4o75	-9.6	145	6h5w	-11	94	6ms7	-8.7
153	1hvy	-9.8	128	3lpp	-7.1	153	4o75	-9.5	146	6h5w	-11	96	6ms7	-8.7
144	1hvy	-9.6	26	3lpp	-7.1	146	4o75	-9.3	149	6h5w	-11	112	6ms7	-8.6

143	1hw8	-10.7	127	3lpp	-7	148	4o75	-9.3	142	6h5w	-10.9	113	6ms7	-8.6
142	1hw8	-10.6	32	3lpp	-7	115	4o75	-9.2	153	6h5w	-10.9	114	6ms7	-8.6
151	1hw8	-10.5	92	3lpp	-7	122	4o75	-9.2	152	6h5w	-10.7	116	6ms7	-8.6
152	1hw8	-10.5	96	3lpp	-7	147	4o75	-9	148	6h5w	-10.3	122	6ms7	-8.6
150	1hw8	-10.4	108	3lpp	-6.9	110	4o75	-8.9	116	6h5w	-8.9	129	6ms7	-8.6
153	1hw8	-10.1	134	3lpp	-6.9	85	4o75	-8.9	126	6h5w	-8.9	135	6ms7	-8.6
147	1hw8	-10	27	3lpp	-6.9	109	4o75	-8.8	141	6h5w	-8.9	84	6ms7	-8.6
148	1hw8	-10	97	3lpp	-6.9	98	4o75	-8.8	112	6h5w	-8.8	86	6ms7	-8.6
144	1hw8	-9.9	109	3lpp	-6.8	100	4o75	-8.7	129	6h5w	-8.8	97	6ms7	-8.6
146	1hw8	-9.9	138	3lpp	-6.8	104	4o75	-8.7	135	6h5w	-8.8	98	6ms7	-8.6
145	1hw8	-9.8	140	3lpp	-6.8	107	4o75	-8.7	106	6h5w	-8.7	104	6ms7	-8.5
149	1hw8	-9.8	141	3lpp	-6.8	108	4o75	-8.7	107	6h5w	-8.7	106	6ms7	-8.5
141	1hw8	-8.5	31	3lpp	-6.8	111	4o75	-8.7	109	6h5w	-8.7	110	6ms7	-8.5
135	1hw8	-8.3	35	3lpp	-6.8	113	4o75	-8.7	115	6h5w	-8.7	115	6ms7	-8.5
115	1hw8	-8.2	67	3lpp	-6.8	83	4o75	-8.7	139	6h5w	-8.7	121	6ms7	-8.5
125	1hw8	-8.2	153	3lpp	-6.7	114	4o75	-8.6	85	6h5w	-8.7	123	6ms7	-8.5
134	1hw8	-8.2	22	3lpp	-6.7	121	4o75	-8.6	97	6h5w	-8.7	136	6ms7	-8.5
98	1hw8	-8.2	24	3lpp	-6.7	92	4o75	-8.6	100	6h5w	-8.6	149	6ms7	-8.5
111	1hw8	-8.1	43	3lpp	-6.7	93	4o75	-8.6	102	6h5w	-8.6	83	6ms7	-8.5
124	1hw8	-8.1	53	3lpp	-6.7	99	4o75	-8.6	111	6h5w	-8.6	91	6ms7	-8.5

137	1hw8	-8.1	90	3lpp	-6.7	105	4o75	-8.5	120	6h5w	-8.6	95	6ms7	-8.5
138	1hw8	-8.1	74	3lpp	-6.6	119	4o75	-8.5	122	6h5w	-8.6	99	6ms7	-8.5
109	1hw8	-8	101	3lpp	-6.5	120	4o75	-8.5	125	6h5w	-8.6	111	6ms7	-8.4
110	1hw8	-8	14	3lpp	-6.5	137	4o75	-8.5	83	6h5w	-8.6	128	6ms7	-8.4
120	1hw8	-8	25	3lpp	-6.5	91	4o75	-8.5	89	6h5w	-8.6	131	6ms7	-8.4
126	1hw8	-8	46	3lpp	-6.5	94	4o75	-8.5	95	6h5w	-8.6	125	6ms7	-8.3
129	1hw8	-8	21	3lpp	-6.4	95	4o75	-8.5	96	6h5w	-8.6	92	6ms7	-8.3
139	1hw8	-8	36	3lpp	-6.4	101	4o75	-8.4	98	6h5w	-8.6	93	6ms7	-8.3
91	1hw8	-8	41	3lpp	-6.4	130	4o75	-8.4	103	6h5w	-8.5	103	6ms7	-8.1
100	1hw8	-7.9	68	3lpp	-6.4	136	4o75	-8.4	108	6h5w	-8.5	153	6rz4	-13.1
104	1hw8	-7.9	16	3lpp	-6.3	96	4o75	-8.4	110	6h5w	-8.5	150	6rz4	-12.9
121	1hw8	-7.9	29	3lpp	-6.3	102	4o75	-8.3	114	6h5w	-8.5	152	6rz4	-12.9
128	1hw8	-7.9	30	3lpp	-6.3	103	4o75	-8.3	118	6h5w	-8.5	130	6x3t	-8.7
136	1hw8	-7.9	33	3lpp	-6.3	117	4o75	-8.3	124	6h5w	-8.5	133	6x3t	-8.7
140	1hw8	-7.9	40	3lpp	-6.3	133	4o75	-8.3	128	6h5w	-8.5	82	6x3t	-8.4
85	1hw8	-7.9	48	3lpp	-6.3	138	4o75	-8.3	137	6h5w	-8.5	114	6x3t	-8.3
94	1hw8	-7.9	57	3lpp	-6.3	97	4o75	-8.3	140	6h5w	-8.5	117	6x3t	-8.3
101	1hw8	-7.8	18	3lpp	-6.2	112	4o75	-8.2	88	6h5w	-8.5	122	6x3t	-8.3
112	1hw8	-7.8	34	3lpp	-6.2	118	4o75	-8.2	151	6jb3	-10.8	123	6x3t	-8.3
114	1hw8	-7.8	51	3lpp	-6.2	141	4o75	-8.2	153	6jb3	-10.5	17	6x3t	-8.3
116	1hw8	-7.8	76	3lpp	-6.2	87	4o75	-8.2	152	6jb3	-	102	6x3t	-8.2

10.4

118	1hw8	-7.8	13	3lpp	-6.1	89	4o75	-8.2	144	6jb3	-10.3	104	6x3t	-8.2
122	1hw8	-7.8	17	3lpp	-6.1	90	4o75	-8.2	142	6jb3	-10.1	107	6x3t	-8.2
123	1hw8	-7.8	20	3lpp	-6.1	132	4o75	-8.1	143	6jb3	-10.1	113	6x3t	-8.2
88	1hw8	-7.8	23	3lpp	-6.1	67	4o75	-8.1	145	6jb3	-10.1	129	6x3t	-8.2
89	1hw8	-7.8	37	3lpp	-6.1	82	4o75	-8.1	147	6jb3	-10.1	138	6x3t	-8.2
107	1hw8	-7.7	55	3lpp	-6.1	84	4o75	-8.1	150	6jb3	-10.1	84	6x3t	-8.2
108	1hw8	-7.7	77	3lpp	-6.1	86	4o75	-8.1	148	6jb3	-9.7	98	6x3t	-8.2
127	1hw8	-7.7	7	3lpp	-6.1	106	4o75	-8	149	6jb3	-9.6	108	6x3t	-8.1
131	1hw8	-7.7	148	3pix	-9.9	116	4o75	-8	146	6jb3	-9.5	121	6x3t	-8.1
84	1hw8	-7.7	149	3pix	-9.8	128	4o75	-8	141	6jb3	-8.3	83	6x3t	-8.1
92	1hw8	-7.7	151	3pix	-9.7	88	4o75	-8	130	6jb3	-8.2	118	6x3t	-8
95	1hw8	-7.7	152	3pix	-9.7	129	4o75	-7.9	133	6jb3	-8.2	137	6x3t	-8
99	1hw8	-7.7	145	3pix	-9.6	123	4o75	-7.8	132	6jb3	-8.1	140	6x3t	-8
57	1hw8	-7.6	147	3pix	-9.6	125	4o75	-7.8	92	6jb3	-8.1	43	6x3t	-8
83	1hw8	-7.6	144	3pix	-9.5	126	4o75	-7.8	122	6jb3	-8	85	6x3t	-8
93	1hw8	-7.6	153	3pix	-9.5	131	4o75	-7.8	126	6jb3	-8	86	6x3t	-8
105	1hw8	-7.5	142	3pix	-9.4	134	4o75	-7.7	108	6jb3	-7.9	93	6x3t	-8
113	1hw8	-7.5	146	3pix	-9.4	135	4o75	-7.7	128	6jb3	-7.9	99	6x3t	-8
119	1hw8	-7.5	150	3pix	-9.4	57	4o75	-7.7	138	6jb3	-7.9	105	6x3t	-7.9

130	1hw8	-7.5	143	3pix	-9.3	150	5btr	-11.8	140	6jb3	-7.9	106	6x3t	-7.9
132	1hw8	-7.5	120	3pix	-8.6	151	5btr	-11.5	98	6jb3	-7.9	110	6x3t	-7.9
133	1hw8	-7.5	141	3pix	-8.6	142	5btr	-10.7	101	6jb3	-7.8	125	6x3t	-7.9
96	1hw8	-7.5	108	3pix	-8.5	147	5btr	-10.7	117	6jb3	-7.8	128	6x3t	-7.9
103	1hw8	-7.4	115	3pix	-8.5	144	5btr	-10.6	119	6jb3	-7.8	19	6x3t	-7.9
106	1hw8	-7.4	95	3pix	-8.5	146	5btr	-10.5	135	6jb3	-7.8	41	6x3t	-7.9
117	1hw8	-7.4	97	3pix	-8.5	148	5btr	-10.5	139	6jb3	-7.8	96	6x3t	-7.9
82	1hw8	-7.4	98	3pix	-8.4	149	5btr	-10.5	112	6jb3	-7.7	97	6x3t	-7.9
86	1hw8	-7.4	106	3pix	-8.3	143	5btr	-10.3	113	6jb3	-7.7	119	6x3t	-7.8
142	1us0	-12.4	99	3pix	-8.3	152	5btr	-10.3	114	6jb3	-7.7	126	6x3t	-7.8
151	1us0	-12.2	105	3pix	-8.2	145	5btr	-10.2	115	6jb3	-7.7	59	6x3t	-7.8
143	1us0	-12	107	3pix	-8.2	153	5btr	-9.6	125	6jb3	-7.7	66	6x3t	-7.8
147	1us0	-12	112	3pix	-8.2	141	5btr	-9.2	127	6jb3	-7.7	88	6x3t	-7.8
149	1us0	-11.9	113	3pix	-8.2	108	5btr	-9	131	6jb3	-7.7	89	6x3t	-7.8

144	1us0	-11.8	126	3pix	-8.2	117	5btr	-8.8	134	6jb3	-7.7	92	6x3t	-7.8
150	1us0	-11.8	136	3pix	-8.2	82	5btr	-8.8	136	6jb3	-7.7	95	6x3t	-7.8
145	1us0	-11.7	85	3pix	-8.2	83	5btr	-8.8	137	6jb3	-7.7	100	6x3t	-7.7
148	1us0	-11.7	110	3pix	-8.1	102	5btr	-8.7	88	6jb3	-7.7	116	6x3t	-7.7
108	1us0	-11.1	114	3pix	-8.1	118	5btr	-8.7	89	6jb3	-7.7	90	6x3t	-7.7
82	1us0	-10.9	122	3pix	-8.1	93	5btr	-8.7	94	6jb3	-7.7	91	6x3t	-7.7
83	1us0	-10.9	125	3pix	-8.1	104	5btr	-8.6	95	6jb3	-7.7	94	6x3t	-7.7
113	1us0	-10.8	128	3pix	-8.1	105	5btr	-8.6	99	6jb3	-7.7	131	6x3t	-7.6
117	1us0	-10.8	91	3pix	-8.1	107	5btr	-8.6	104	6jb3	-7.6	20	6x3t	-7.6
93	1us0	-10.8	93	3pix	-8.1	113	5btr	-8.6	111	6jb3	-7.6	48	6x3t	-7.6
99	1us0	-10.8	143	3roc	-11.5	114	5btr	-8.6	118	6jb3	-7.6	70	6x3t	-7.6
102	1us0	-10.7	147	3roc	-11.5	137	5btr	-8.6	120	6jb3	-7.6	73	6x3t	-7.6
105	1us0	-10.7	142	3roc	-11.4	85	5btr	-8.6	124	6jb3	-7.6	103	6x3t	-7.5

85	1us0	-10.7	151	3roc	-11.2	87	5btr	-8.6	87	6jb3	-7.6	112	6x3t	-7.5
104	1us0	-10.6	144	3roc	-11.1	109	5btr	-8.5	91	6jb3	-7.6	115	6x3t	-7.5
114	1us0	-10.6	150	3roc	-11	110	5btr	-8.5	93	6jb3	-7.6	132	6x3t	-7.5
121	1us0	-10.6	148	3roc	-10.9	119	5btr	-8.5	102	6jb3	-7.5	139	6x3t	-7.5
122	1us0	-10.6	145	3roc	-10.8	138	5btr	-8.5	105	6jb3	-7.5	55	6x3t	-7.5
146	1us0	-10.6	146	3roc	-10.8	84	5btr	-8.5	106	6jb3	-7.5	87	6x3t	-7.5
84	1us0	-10.6	149	3roc	-10.7	86	5btr	-8.5	129	6jb3	-7.5	101	6x3t	-7.4
98	1us0	-10.6	152	3roc	-10.7	88	5btr	-8.5	83	6jb3	-7.5	109	6x3t	-7.4
111	1us0	-10.5	153	3roc	-10.4	90	5btr	-8.5	85	6jb3	-7.5	127	6x3t	-7.4
119	1us0	-10.5	122	3roc	-9.6	94	5btr	-8.5	97	6jb3	-7.5	124	6x3t	-7.3
103	1us0	-10.4	124	3roc	-9.6	98	5btr	-8.5	103	6jb3	-7.4	134	6x3t	-7.3
110	1us0	-10.4	147	4mdd	-10.6	99	5btr	-8.5	107	6jb3	-7.4	136	6x3t	-7.3
118	1us0	-10.4	142	4mdd	-10.5	106	5btr	-8.4	121	6jb3	-7.4	14	6x3t	-7.3

123	1us0	-10.4	143	4mdd	-10.4	121	5btr	-8.4	123	6jb3	-7.4	1	6x3t	-7.3
137	1us0	-10.4	151	4mdd	-10.3	122	5btr	-8.4	36	6jb3	-7.4	22	6x3t	-7.3
86	1us0	-10.4	144	4mdd	-10.2	89	5btr	-8.4	82	6jb3	-7.4	40	6x3t	-7.3
92	1us0	-10.4	145	4mdd	-9.9	95	5btr	-8.4	84	6jb3	-7.4	67	6x3t	-7.3
107	1us0	-10.3	149	4mdd	-9.8	100	5btr	-8.3	100	6jb3	-7.3	68	6x3t	-7.3
116	1us0	-10.3	152	4mdd	-9.8	111	5btr	-8.3	109	6jb3	-7.3	79	6x3t	-7.3
87	1us0	-10.3	153	4mdd	-9.7	116	5btr	-8.3	110	6jb3	-7.3	141	6x3t	-7.2
88	1us0	-10.3	146	4mdd	-9.4	91	5btr	-8.3	116	6jb3	-7.3	15	6x3t	-7.2
101	1us0	-10.2	148	4mdd	-9.3	96	5btr	-8.3	86	6jb3	-7.3	21	6x3t	-7.2
106	1us0	-10.2	150	4mdd	-9.2	115	5btr	-8.2	82	6kxx	-9.8	23	6x3t	-7.2
109	1us0	-10.2	99	4mdd	-8.8	120	5btr	-8.2	118	6kxx	-9.7	75	6x3t	-7.2
139	1us0	-10.2	113	4mdd	-8.7	130	5btr	-8.2	137	6kxx	-9.5	111	6x3t	-7.1
95	1us0	-10.2	122	4mdd	-8.7	97	5btr	-8.2	141	6m9t	-10.5	120	6x3t	-7.1

112	1us0	-10.1	104	4mdd	-8.6	123	5btr	-8.1	119	6m9t	-10.2	18	6x3t	-7.1
115	1us0	-10.1	109	4mdd	-8.6	125	5btr	-8.1	101	6m9t	-10	3	6x3t	-7.1
120	1us0	-10.1	105	4mdd	-8.5	127	5btr	-8.1	94	6m9t	-10	45	6x3t	-7.1
124	1us0	-10.1	107	4mdd	-8.5	128	5btr	-8.1	97	6m9t	-10	58	6x3t	-7.1
131	1us0	-10.1	115	4mdd	-8.5	133	5btr	-8.1	99	6m9t	-10	16	6x3t	-7
135	1us0	-10.1	117	4mdd	-8.5	112	5btr	-8	108	6m9t	-9.9	74	6x3t	-7
138	1us0	-10.1	100	4mdd	-8.4	126	5btr	-8	109	6m9t	-9.9	7	6x3t	-7
89	1us0	-10.1	102	4mdd	-8.4	129	5btr	-8	121	6m9t	-9.9	13	6x3t	-6.9
90	1us0	-10.1	116	4mdd	-8.4	135	5btr	-8	122	6m9t	-9.9	27	6x3t	-6.9
94	1us0	-10.1	83	4mdd	-8.4	140	5btr	-8	138	6m9t	-9.9	34	6x3t	-6.9
100	1us0	-10	88	4mdd	-8.4	57	5btr	-8	151	6m9t	-9.9	6	6x3t	-6.9
130	1us0	-10	90	4mdd	-8.4	124	5btr	-7.9	90	6m9t	-9.9	36	6x3t	-6.8
132	1us0	-10	93	4mdd	-8.4	136	5btr	-7.9	96	6m9t	-9.9	4	6x3t	-6.8
133	1us0	-10	97	4mdd	-8.4	139	5btr	-7.9	98	6m9t	-9.9	28	6x3t	-6.7
96	1us0	-10	111	4mdd	-8.3	101	5btr	-7.8	100	6m9t	-9.8	30	6x3t	-6.7
97	1us0	-10	137	4mdd	-8.3	103	5btr	-7.8	111	6m9t	-9.8	38	6x3t	-6.7

141	1us0	-9.9	85	4mdd	-8.3	132	5btr	-7.8	113	6m9t	-9.8	47	6x3t	-6.7
91	1us0	-9.9	86	4mdd	-8.3	134	5btr	-7.8	115	6m9t	-9.8	69	6x3t	-6.7
21	3ctt	-7.3	91	4mdd	-8.3	92	5btr	-7.8	120	6m9t	-9.8	78	6x3t	-6.7
24	3ctt	-7.2	92	4mdd	-8.3	131	5btr	-7.7	125	6m9t	-9.8	150	5ISO	-9.3
41	3ctt	-6.9	110	4mdd	-8.2	55	5btr	-7.2	126	6m9t	-9.8	151	5ISO	-9.2
30	3ctt	-6.8	112	4mdd	-8.2	81	5btr	-7.2	137	6m9t	-9.8	101	5ISO	-8.8
33	3ctt	-6.6	114	4mdd	-8.2	124	5h8q	-10.7	86	6m9t	-9.8	102	5ISO	-8.8
102	3ctt	-6.5	118	4mdd	-8.2	138	5h8q	-10.7	87	6m9t	-9.8	113	5ISO	-8.8
82	3ctt	-6.5	121	4mdd	-8.2	137	5h8q	-10.6	92	6m9t	-9.8	114	5ISO	-8.8
116	3ctt	-6.4	141	4mdd	-8.2	142	5i6x	-10.9	95	6m9t	-9.8	122	5ISO	-8.8
88	3ctt	-6.4	82	4mdd	-8.2	147	5i6x	-10.8	106	6m9t	-9.7	84	5ISO	-8.8
89	3ctt	-6.4	94	4mdd	-8.2	151	5i6x	-10.8	107	6m9t	-9.7	91	5ISO	-8.8
118	3ctt	-6.3	96	4mdd	-8.2	143	5i6x	-10.7	129	6m9t	-9.7	98	5ISO	-8.8
43	3ctt	-6.3	98	4mdd	-8.2	122	5i6x	-10.4	140	6m9t	-9.7	107	5ISO	-8.7
10	3ctt	-6.1	106	4mdd	-8.1	118	5ikr	-9.2	85	6m9t	-9.7	108	5ISO	-8.7
122	3ctt	-6.1	108	4mdd	-8.1	82	5ikr	-9.1	89	6m9t	-9.7	115	5ISO	-8.7
7	3ctt	-6.1	119	4mdd	-8.1	150	6g54	-10.1	91	6m9t	-9.7	117	5ISO	-8.7

115	3ctt	-6	87	4mdd	-8.1	147	6g54	-9.8	105	6m9t	-9.6	121	5ISO	-8.7
34	3ctt	-6	95	4mdd	-8.1	152	6g54	-9.8	110	6m9t	-9.6	136	5ISO	-8.7
83	3ctt	-6	103	4mdd	-8	153	6g54	-9.8	114	6m9t	-9.6	142	5ISO	-8.7
138	3ctt	-5.9	120	4mdd	-8	151	6g54	-9.7	117	6m9t	-9.6	82	5ISO	-8.7
16	3ctt	-5.9	124	4mdd	-8	143	6g54	-9.6	118	6m9t	-9.6	83	5ISO	-8.7
112	3ctt	-5.8	130	4mdd	-8	142	6g54	-9.5	130	6m9t	-9.6	85	5ISO	-8.7
128	3ctt	-5.8	133	4mdd	-8	145	6g54	-9.5	131	6m9t	-9.6	95	5ISO	-8.7
137	3ctt	-5.8	136	4mdd	-8	149	6g54	-9.5	133	6m9t	-9.6	106	5ISO	-8.6
140	3ctt	-5.8	139	4mdd	-8	146	6g54	-9.4	88	6m9t	-9.6	109	5ISO	-8.6
22	3ctt	-5.8	84	4mdd	-8	148	6g54	-9.4	103	6m9t	-9.5	112	5ISO	-8.6
9	3ctt	-5.8	89	4mdd	-8	144	6g54	-9.3	128	6m9t	-9.5	143	5ISO	-8.6
74	3ctt	-5.7	123	4mdd	-7.9	141	6g54	-8.7	134	6m9t	-9.5	147	5ISO	-8.6
85	3ctt	-5.7	138	4mdd	-7.9	128	6g54	-8.4	135	6m9t	-9.5	94	5ISO	-8.6
142	3lpp	-9.5	101	4mdd	-7.8	111	6g54	-8.3	136	6m9t	-9.5	99	5ISO	-8.6
144	3lpp	-9.4	131	4mdd	-7.6	122	6g54	-8.3	84	6m9t	-9.5	103	5ISO	-8.5
149	3lpp	-9.2	41	4mdd	-7.6	125	6g54	-8.3	102	6m9t	-9.4	105	5ISO	-8.5
151	3lpp	-9.1	125	4mdd	-7.5	112	6g54	-8.2	112	6m9t	-9.4	118	5ISO	-8.5
145	3lpp	-9	128	4mdd	-7.5	120	6g54	-8.2	124	6m9t	-9.4	134	5ISO	-8.5
147	3lpp	-9	132	4mdd	-7.5	126	6g54	-8.2	127	6m9t	-9.4	139	5ISO	-8.5
150	3lpp	-9	140	4mdd	-7.5	97	6g54	-8.2	57	6m9t	-9.4	144	5ISO	-8.5
143	3lpp	-8.8	43	4mdd	-7.5	135	6g54	-8.1	93	6m9t	-9.4	100	5ISO	-8.4
123	3lpp	-8	10	4mdd	-7.4	92	6g54	-8.1	104	6m9t	-9.3	104	5ISO	-8.4
118	3lpp	-7.9	126	4mdd	-7.4	108	6g54	-8	123	6m9t	-9.3	123	5ISO	-8.4
130	3lpp	-7.9	129	4mdd	-7.4	110	6g54	-8	82	6m9t	-9.2	129	5ISO	-8.4
133	3lpp	-7.9	49	4mdd	-7.4	115	6g54	-8	83	6m9t	-9.2	130	5ISO	-8.4
137	3lpp	-7.9	57	4mdd	-7.4	129	6g54	-8	116	6m9t	-9.1	133	5ISO	-8.4

146	3lpp	-7.9	61	4mdd	-7.4	130	6g54	-8	139	6m9t	-9.1	86	5ISO	-8.4
82	3lpp	-7.9	72	4mdd	-7.4	133	6g54	-8	19	6m9t	-9	93	5ISO	-8.4
91	3lpp	-7.9	127	4mdd	-7.3	57	6g54	-8	2	6m9t	-9	150	6MD4	-11.4
117	3lpp	-7.8	134	4mdd	-7.3	85	6g54	-8	64	6m9t	-8.9	142	6MD4	-11.2
88	3lpp	-7.8	135	4mdd	-7.3	102	6g54	-7.9	132	6m9t	-8.8	151	6MD4	-11.2
104	3lpp	-7.7	28	4mdd	-7.3	104	6g54	-7.9	48	6m9t	-8.8	143	6MD4	-10.9
120	3lpp	-7.7	37	4mdd	-7.3	109	6g54	-7.9	147	6ms7	-12.3	152	6MD4	-10.9
122	3lpp	-7.7	122	4o1z	-9.6	114	6g54	-7.9	152	6ms7	-11.5	149	6MD4	-10.8
136	3lpp	-7.7	82	4o1z	-9.6	127	6g54	-7.9	151	6ms7	-11.1	153	6MD4	-10.8
28	3lpp	-7.7	83	4o1z	-9.6	136	6g54	-7.9	153	6ms7	-11.1	148	6MD4	-10.6
83	3lpp	-7.7	114	4o1z	-9.5	140	6g54	-7.9	150	6ms7	-10.9	144	6MD4	-10.4
94	3lpp	-7.7	93	4o1z	-9.5	75	6g54	-7.9	144	6ms7	-10.6	147	6MD4	-10.4
111	3lpp	-7.6	100	4o1z	-9.4	81	6g54	-7.9	145	6ms7	-10.6	145	6MD4	-10.2
115	3lpp	-7.6	102	4o1z	-9.4	91	6g54	-7.9	146	6ms7	-10.6	146	6MD4	-10.2

116	3lpp	-7.6	110	4o1z	-9.4	95	6g54	-7.9	142	6ms7	-10.2	141	6MD4	-9
124	3lpp	-7.6	98	4o1z	-9.4	98	6g54	-7.9	143	6ms7	-9.6	108	6MD4	-8.9
84	3lpp	-7.6	104	4o1z	-9.3	99	6g54	-7.9	118	6ms7	-9.5	118	6MD4	-8.9
93	3lpp	-7.6	117	4o1z	-9.3	100	6g54	-7.8	137	6ms7	-9.4	120	6MD4	-8.9
105	3lpp	-7.5	118	4o1z	-9.3	106	6g54	-7.8	139	6ms7	-9.4	89	6MD4	-8.9
113	3lpp	-7.5	137	4o1z	-9.3	107	6g54	-7.8	141	6ms7	-9.3	91	6MD4	-8.9
121	3lpp	-7.5	88	4o1z	-9.3	121	6g54	-7.8	127	6ms7	-9.1	102	6MD4	-8.8
131	3lpp	-7.5	107	4o1z	-9.2	124	6g54	-7.8	82	6ms7	-9.1	109	6MD4	-8.8
132	3lpp	-7.5	111	4o1z	-9.2	131	6g54	-7.8	108	6ms7	-9	111	6MD4	-8.8
87	3lpp	-7.5	134	4o1z	-9.2	88	6g54	-7.8	134	6ms7	-8.9	119	6MD4	-8.8
102	3lpp	-7.4	84	4o1z	-9.2	89	6g54	-7.8	138	6ms7	-8.9	88	6MD4	-8.8
107	3lpp	-7.4	105	4o1z	-9.1	93	6g54	-7.8	101	6ms7	-8.8	100	6MD4	-8.7
114	3lpp	-7.4	121	4o1z	-9.1	101	6g54	-7.7	109	6ms7	-8.8	106	6MD4	-8.7
139	3lpp	-7.4	85	4o1z	-9.1	103	6g54	-7.7	124	6ms7	-8.8	110	6MD4	-8.7
95	3lpp	-7.4	92	4o1z	-9.1	105	6g54	-7.7	130	6ms7	-8.8	112	6MD4	-8.7
100	3lpp	-7.3	103	4o1z	-9	113	6g54	-7.7	133	6ms7	-8.8	114	6MD4	-8.7
119	3lpp	-7.3	109	4o1z	-9	116	6g54	-7.7	87	6ms7	-8.8	117	6MD4	-8.7
135	3lpp	-7.3	113	4o1z	-9	134	6g54	-7.7	90	6ms7	-8.8	129	6MD4	-8.7
148	3lpp	-7.3	116	4o1z	-9	137	6g54	-7.7	100	6ms7	-8.7	136	6MD4	-8.7
152	3lpp	-7.3	123	4o1z	-9	19	6g54	-7.7	102	6ms7	-8.7	84	6MD4	-8.7
98	3lpp	-7.3	124	4o1z	-9	29	6g54	-7.7	105	6ms7	-8.7	86	6MD4	-8.7
99	3lpp	-7.3	127	4o1z	-9	37	6g54	-7.7	107	6ms7	-8.7	95	6MD4	-8.7
